# Fishers and groupers (*Epinephelus marginatus* and *E. morio)* in the coast of Brazil: integrating information for conservation

**DOI:** 10.1186/s13002-019-0331-2

**Published:** 2019-11-06

**Authors:** Alpina Begossi, Svetlana Salyvonchyk, Branko Glamuzina, Shirley Pacheco de Souza, Priscila F. M. Lopes, Regina H. G. Priolli, Djalma Osmanir do Prado, Milena Ramires, Mariana Clauzet, Cleverson Zapelini, Daiana T. Schneider, Luis T. Silva, Renato A. M. Silvano

**Affiliations:** 1 Fisheries and Food Institute, FIFO (www.fisheriesandfood.com), Santos, Brazil; 2 Nepa, Capesca, UNICAMP, Av. Albert Einstein 291, Campinas, SP CEP: 13083-852 Brazil; 3 PPG Ecomar, UNISANTA, R. Cesário Mota 08, Santos, SP CEP: 11045-040 Brazil; 4 Institute for Nature Management National Academy of Sciences of Belarus, 10 Skaryna Street, 220114 Minsk, Belarus; 5 Department of Aquaculture, University of Dubrovnik, 20207 Dubrovnik, Croatia; 6 Federal Institute of Education, Science and Technology of São Paulo, Caraguatatuba, SP 11667 Brazil; 7 Department of Ecology, Fishing Ecology, Management, and Economics group, Federal University of Rio Grande do Norte, Natal, RN 59078-900 Brazil; 8 PPED, IE, UFRJ, Av. Pasteur 250, Rio de Janeiro, RJ CEP: 22.290-902 Brazil; 9 Ethnoconservation and Protected Areas Lab (LECAP), Collaborator researcher of Programa de Pós-Graduação em Sistemas Aquáticos Tropicais, Universidade Estadual de Santa Cruz, Ilhéus, BA Brazil; 10 Department of Ecology, Federal University of Rio Grande do Sul, UFRGS, CP:15007, Porto Alegre, RS CEP: 91501-970 Brazil

**Keywords:** Local knowledge, Fisheries management, Small-scale fisheries, Endangered species

## Abstract

**Background:**

Groupers are a vulnerable but economically important group of fish, especially for small-scale fisheries. We investigated catches and local ecological knowledge (LEK) of diet, habitat, and past fishing experiences.

**Methods:**

Landings, prices, interviews, and restaurants demand for two species, *Epinephelus marginatus* (dusky grouper) and *Epinephelus morio* (red grouper), were registered.

**Results:**

We visited 74 markets and 79 sites on the coast of Brazil in 2017–2018, and we interviewed 71 fishers: Bahia (NE), Rio de Janeiro and São Paulo (SE), and Santa Catarina (S). The landings sampled of dusky grouper (2016–2017) in Rio de Janeiro were: *n* = 222, size 38–109 cm, weight 1–24 kg, average 3.84 kg; in São Paulo, São Sebastião were: *n* = 47, size 39–106 cm, weight 2–8 kg, average of 2.77 kg; and at Santos: *n* = 80, 26–120 cm, weight 0.36–15 kg, average 2.72 kg. Red grouper was observed in markets in the northeastern Brazil. We did not observe *Epinephelus marginatus* from Bahia northward; a maximum size of 200 cm was reported south of the Bahia, besides Rio de Janeiro and São Paulo coasts, 20 years ago (or longer) by 12 fishers. Local knowledge of fishers was important for grouper data of habitat and diet; the reproduction period was identified by fishers as September to March.

**Conclusions:**

Groupers can be considered as a cultural and ecological keystone species. We suggest protective measures: 1) fishing zoning, 2) islands (MPAs) with the surveillance of fishers, 3) late Spring and early Summer as key periods for management (grouper reproduction), 4) studies on grouper larvae, 5) mapping of fishing spots, 6) studies on local knowledge. Collaboration with small-scale fishers and local knowledge could contribute to low-conflict management measures. In that regard, integrative models of management from Latin America, by using local knowledge and citizen science, could produce successful grouper management for Brazilian data-poor fisheries, a contrasting reality to the Mediterranean areas. Finally, the distribution of *E. marginatus* in Brazil leave us with questions: a) Have dusky groupers disappeared from Bahia because of a decline in the population? b) Was it uncommon in Northeast Brazil? c) Did changes in water temperatures forced a movement southward?

## Background

Several marine fish species that play significant ecological and cultural roles are globally threatened by overfishing, pollution and possibly climate change. The conservation of marine ecosystems faces many challenges [1] that are related to the characteristics of the local species. In particular, groupers have previously been reported to be impacted by professional (including small-scale fisheries) and recreational fishing techniques.

Groupers are large, sedentary fish that are slow-growing. Groupers are often caught by fishers using spears or hooks and lines. Despite the economic importance of groupers, small-scale fisheries off the coast of Brazil have conflicting interests, and little attention has been paid to the proper management of this industry [2]. However, there are many examples of dusky grouper management in the Mediterranean Sea, including the use of marine protected areas and the critical aspect of larval dispersion (MPAs) [3–6].

In Brazil, the catches from small-scale fisheries represent more than half of the total national fish production [7], and noble and prized fish species, such as groupers (Epinephelidae), are targeted by small-scale fisheries [2, 8].

However, the management of these small-scale fisheries is either ignored or conflicts with the livelihoods of local fishers were observed [9, 10]. Most artisanal fishers are poor individuals whose livelihoods depend on fish, and these individuals are often in conflict with the government’s environmental officers [9–15], especially through the top-down establishment of MPAs. The conflicts between MPAs and local fishers, although difficult to address, can be solved by including the fishers in the planning, implementation and functioning of the MPAs [12, 13]. In particular, some artisanal fishers in Brazil live in remote areas, such as fishing communities along the Atlantic Forest coast, while others operate in highly urbanized areas, such as Rio de Janeiro. The most recent Brazilian population census, which was conducted in 2010, reported that 84% of the Brazilian population lived in urban areas [16]. However, despite the several social, economic and environmental impacts of urbanization on the communities of small-scale fishers, these individuals comprise an important socioeconomic group. In highly urbanized coastal states, such as Rio de Janeiro and São Paulo, small municipalities with low levels of urbanization have many small-scale fishers. Moreover, small-scale fishers account for more than 26% of the total population even in the highly urbanized areas of these states [17]. In addition, most areas of the Atlantic Forest coast are visited by tourists year-round, and small-scale fishers are part of the regional economic market [10, 18].

Thus, the dusky grouper [garoupa, in Brazilian Portuguese; cernia in Italian; mero in Portuguese and Spanish], *Epinephelus marginatus,* is a fish that is important for the livelihoods of small-scale fishers on the Brazilian Atlantic coast (the popular name mero in Brazil is another species, *E. itajara*). Dusky grouper is a preferred food by fishers and has high market prices [8, 19]. This species is a protogynous, monandric hermaphrodite reef fish that is distributed throughout the Atlantic Ocean, including the coast of Brazil, the Mediterranean Sea, and the African coast [20, 21]. The species has high longevity and a slow growth rate; however, its aggregated spawning behavior makes it vulnerable to fishing pressure. *E. morio* is found on the coast of the USA, in the Caribbean and Brazil [22–24]. *E. morio* is a protogynous hermaphrodite with slow growth rates and late maturity, and it likely forms seasonal spawning aggregations [25–29]; such features make this species more sensitive because fishing can affect the abundance of males in the population [30].

*E. marginatus* has been classified as endangered on the IUCN Red List [31], which is especially worrisome given its ecological importance [21]. Fennessy [32, 33] considered overexploitation the major threat for *E. marginatus*, since its slow growth, protogynous hermaphroditism and spawning aggregation behaviour render it vulnerable to fishing pressures.

Although much is known about this species in the Mediterranean, information about this species in Brazil is still scarce. Some studies on this fish have focused on investigating its biology along the southern coast of Brazil [21, 34], while others have focused on investigating its genetics [35], ecology, and fishing patterns via the evaluation of local ecological knowledge (LEK) or collaborative processes with fishers near Southeast Brazil [2, 8, 19, 36, 37]. A comprehensive review of the dusky grouper was recently published by Condini et al. [21], which included an evaluation of the current knowledge of the biology and ecology of this species. Some information that has already been synthesized is as follows [22, 24, 38–45]: groupers (Epinephelidae) includes about 160 species important economically, such as dusky grouper; it is a protogynous hermaphrodite fish, reaching female sexual maturity at 3 kg, with a mean length at first maturity (L50) of 43.8 cm (Ls) and sex reversal occurring at 10 kg [40, 41]. The largest specimens of this fish were caught in Tunisia (35 kg) and in Brazil (60 kg) [40]. Dusky grouper is a solitary and territorial fish with a maximum length of 150 cm, maximum observed age of 50 years and with its distribution in the Atlantic Ocean [22, 24, 42, 43]. This species is very important and high-valued also in the Mediterranean Sea [44, 45].

*E. marginatus* is considered to be comprised of two subpopulations: one in West Africa and Europe and the other in South America [23]. The subpopulation in the Mediterranean experienced a decline of approximately 88% between 1990 and 2001 [23]. This decline was observed even though the Mediterranean Sea includes several MPAs where the dusky grouper is known to occur [5, 41, 46–48].

Given the relative lack of data on groupers in Brazil (especially on the SE Brazilian coast), in this study, we summarized the data available in Brazil and collected data from different categories (landings, local knowledge, among others) on Brazilian groupers from Rio Grande do Norte to Rio Grande do Sul. This type of research is exploratory; when data are scarce, we need to first generate an overview of the study and the questions to be asked to establish future priorities. Contrasted with the Mediterranean, in Brazil, systematic data collection from small-scale fisheries is not mandatory or commonplace. In this regard, exploratory studies are of overwhelming importance and will help us understand why ethnobiological research is so well represented in Brazil, where it serves as a complementary method to acquire biological knowledge. The only recent comprehensive review that is available [21] did not address ethnobiological research or the impact of small-scale fisheries on groupers; instead, it focused on the biology of dusky groupers from different parts of the world, including southern Brazil.

It is also important to stress that there are no management plans for reef species or for the studied grouper species in particular (*E. marginatus* and *E. morio*) in Brazil. An exception to this rule is the very recent *Interministerial Ordinances* (#229, June 27, 2018, and #41, July, 27, 2018), which organize the extraction of *E. marginatus* along with other management procedures, and the *Interministerial Ordinances* (#292, July 18, 2018, and #59_c, November 9, 2018), which include *E. morio* and other species. The management procedures include the establishment of minimum capture sizes and the prohibition of fishing for *E. marginatus* from November 1, 2018, to February 28, 2019. Many authors have scrutinized the unsustainability of government policies regarding the conservation of biodiversity primarily in freshwater systems in Latin America and Brazil; however, these policies have also been applied to marine systems [49–51]. These criticisms include ignoring local knowledge when implementing public policies for conservation, strong economic bias towards private activities, corruption and the observation that many reserves are only paper parks [52].

In studies on the extractive activity of fishing, there might not be a single question but rather an interactive set of questions and multiple approaches. In a study on redfish, Duplisea [53] illustrated an issue in a very straightforward way by showing that information from fishers supported the reinterpretation of population abundance estimates. Another study [54] showed how fishers’ knowledge helped to understand competing explanatory models in fisheries. In particular, the authors showed that fishery management questions should not be shaped as ‘what is the best model’, but rather ‘what should be the management procedures that are more likely to achieve stakeholders’ objectives’ (p. 1287). Lopes et al. [15] used data from both scientific and local knowledge of *Epinephelus marginatus* in Bayesian models to show the importance of local knowledge in predicting species distributions in data-poor fisheries. While it is beyond the scope of this research to provide specific management suggestions, this study will provide data and multilevel, multidisciplinary information (ecology, ethnoecology, ethnobiology, biology) that should aid future management endeavors. To that end, we used the dusky and red grouper as exploratory tools, showing how ethnobiological information may complement our current knowledge.

Our study intends to complement existing data, especially data on dusky grouper in Brazil. Red grouper was included because it is very common and economically important in Northeast Brazil, where no dusky grouper was found. Small-scale fishers in Northeast Brazil refer to red grouper when asked about the dusky grouper. We presented data on dusky grouper in previous studies [2, 8, 19, 35, 38], which focused on other aspects related to small-scale fisheries and LEK. With a focus on Brazil, we summarize selected studies on dusky grouper, present data on this species regarding production by small-scale fisheries in the southeast and present the LEK of groupers from small-scale fisheries along the northeast to the southern coast of the country. We also identify the demand from restaurants and the prices of groupers at selling points. Finally, we discuss suggestions for the management and analyses of these groupers.

## Methods

The procedures in this study included a literature review, systematic collection of fish landing data, interviews, and comparative data analysis of weight-length curves. Systematic monthly data collections (landings) were performed at São Paulo and Rio de Janeiro states; Two trips were done (November 2017 and March 2018) focusing on interviews and visits to markets along the coast of Brazil.

### Literature review

The review was focused especially on Brazil and included the two major groups that have researched *E. marginatus* for 15 years or more. One group conducted biological research in the states of Santa Catarina and Rio Grande do Sul in southern Brazil and was based primarily at Universidade do Vale do Itajaí (A.B. Andrade, M. Hostim-Silva, among others) and Universidade do Rio Grande (M. V. Condini). The second group performed ecological and ethnoecological research in SE Brazil with members of the Fisheries and Food Institute (www.fisheriesandfood.org) (A. Begossi, P. Lopes R.A.M. Silvano, S. Salivonchyk). The interviews that were conducted in early studies are archived at the Fisheries and Food Institute, Unisanta, Santos, SP. Multiple concise tables are provided to facilitate the integration and exposure of data on groupers (Additional file 1: Tables S1–S5).

### Fieldwork

The fieldwork consisted of different steps and objectives, such as visits to fish markets along the coast of Brazil, systematic collection of fish landing data on the SE Brazilian coast, larvae data collection, and conversations with fishers through interviews with fishers.

### Markets

We conducted two major trips to visit markets along the coast of Brazil; other members of the team visited markets on the southern coast. We observed groupers in markets on the coast of Brazil by visiting the areas shown in during two different trips: November 2016 (Bahia coast) and March 2018 (from Rio Grande do Norte to Bahia). During these trips, we spoke with small-scale fishers and utilized guided informal questions as the basis for the informal chats or interviews; through this approach, we acquired information on the LEK nontraditional/nontraditional [55] populations of small-scale fishers.

### Landings

Landings were systematically observed for 3–5 days per month at Copacabana (Rio de Janeiro), São Sebastião and Santos (municipalities of São Paulo state) from 2016 to the beginning of 2018. Landings of dusky groupers were recorded at Copacabana beach, Posto 6, at the box where fishers sell the fish; some records occurred during the process of cleaning it (after the fish is sold). During the same period (2016–2018), fish buyers also voluntarily registered landings of dusky grouper from fishers, even if such purchases occurred outside the sampling dates [2, 38].

### Larvae

We sampled plankton in the main catch area of Copacabana, i.e., the Cagarras Island. Collection trials for larvae were performed on February 17 and 18, 2017. One fisher collaborated with this research project by accompanying us during the transect trials. In two days, 13 trials were performed at Cagarras Island. We used 100 μm plankton nets that were 30 cm in diameter in horizontal and vertical transects; afterward, the samples were conserved in 100 ml of 4% formaldehyde.

### Interviews

We conducted interviews with fishers along the coast of Brazil, at landing sites or at markets, based on structured questions referring to recognition, reproduction (spawning periods) and catch sizes. Interviews were carried out with the fishers that were at the sites, at the moment of our visit. These interviews were complemented, whenever possible, after asking the interviewed to mention other fishers that we should interview. Restaurants were visited to have conversations with administrators and to ask about consumer demands for groupers. We asked about the purchase of groupers by the restaurants, checked the menus and asked how dishes with groupers were prepared. Informal conversations were conducted with fishers along the coast of Brazil, including SE and NE Brazil.

### Weight-length comparisons

**Fig. 1:**
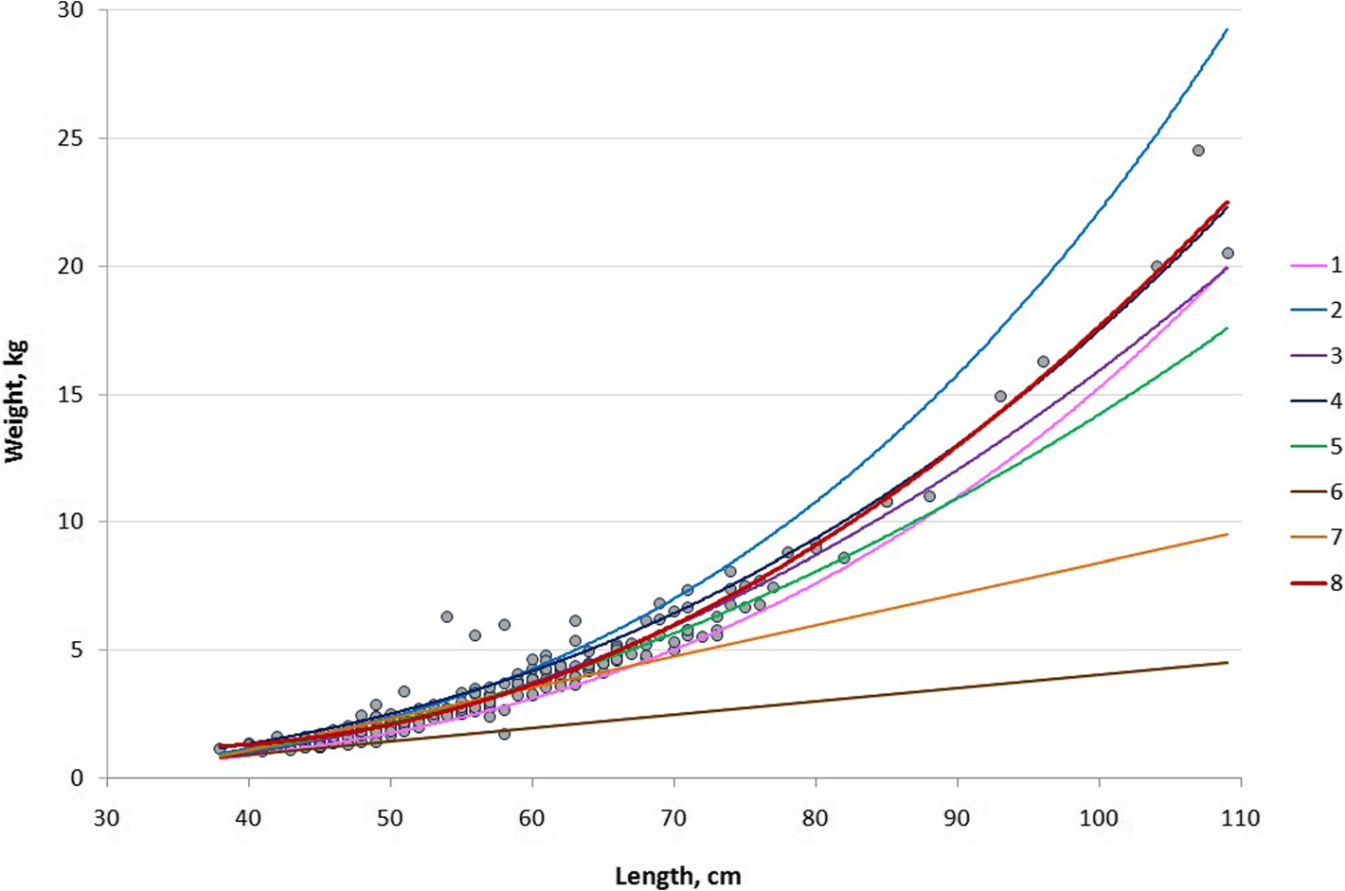
Weight-length of dusky groupers (literature, Table 2). Shown in red are our data from Copacabana from April to November 2016. The other studies are: [1] Andrade et al. [34]; [2] Ximenes-Carvalho et al. [62]; [3] Begossi et al. [2]; [4] Gerhardinger et al. [36, 37]; [5] Lopes et al. [11, 14]; [6] Begossi and Silvano [8]).

We also compared the weight-length curves from the literature. We compared the weight-length curves in Table 1 and Fig. 1 using the root-mean-square deviation (RMSD) and the Kullback-Leibler divergence.

**Table 1:** Selected studies of *E. marginatus* in Brazil (I): growth [Southern Brazil & lab]

Location of interviews/sampling [Total fishers]	Local knowledge on biology and ecology (excluding folk taxonomy)	Reference
Atlantic Forest Coast, Sao Paulo and Southern Rio de Janeiro States) [937]	Cited by 19% as recommended to be eaten during illness	Begossi et al. (2004) [56] (*Ecological Applications*)
Bahia coast, Sao Paulo coast [67]	Habitat and reproduction: 67% said live in reefs/reefs crevices/islands/ 23% reproduce in summer (most do not know) Spawning calendar 54% spring months (Sept., Oct., Nov.) 46% summer months (Dec., Jan., Feb.)	Silvano et al. (2006) [57](*Environ. Biol. Fishes*).
Northern, Southern and South of Brazil (Direct obs RJ and SP) [Set 1 = 92, Set 2 = 49]	Fishing spots (maps) Stomach contents Crabs [65%] and fish [40%] (stomachs not empty = 40) Interviews (set 1) Diet – 19% crustacea, only 4% crabs; 50% fish, mostly sardines; 32% mollusks (*n* = 88) Habitat – 100% reefs, rocks, caves or islands (97% reefs, rocks) (*n* = 88). Interviews (set 2) Diet – 22% crustacea, 12% crabs; 55% fish, mostly sardines; 29% mollusks (*n* = 49) Bait – 45% sardines, 25% bonito, 18% crustaceans, 8% crabs (*n* = 49) Habitat – 96% reefs, rocks, caves or islands (78% reefs, rocks) (*n* = 49) Spawning – 49% do not know; 64% in summer-spring months (*n* = 25) *“Gonads were not macroscopically visible, and we estimated they could be in the category F-1 (resting female) or J-1 (immature females)”.*	Begossi and Silvano (2008) [8] (*Journal of Ethnobiology and Ethnomedicine)*
Paraty, southern coast of Rio de Janeiro State Systematic sampling (*n* = 220)	Stomach contents: 35% crabs, 15% fish and 58% empty (*n* = 220) Fishing spots (maps) Grouper production: 16 months, 220 groupers, 164 kg (4 days / month of sampling)	Begossi et al. (2012, 2014:63 )[19, 58] *Science Journal of Agricultural Research and Management* *2014: Book on Paraty.*
Coast of Brazil. 1986–2009, 14 sites: snappers and groupers	14 fishing communities: a total of 585 fishers were interviewed, 1453 fish were collected, and 1761 fish landings were recorded from 2002 to 2009 (Table, slide)	Begossi et al. (2012) [19] *In: Global Progress in Ecosystem-Based Fisheries Management. Alaska Sea Grant.*
2013–2015 21 months (*n* = 796) Copacabana, Rio (RJ)	Groupers 45-65 cm Fishing spots Diving	Begossi et al. (2016) [2] *J Coast Zone Manag.*
Other studies		
Southern Brazil Arvoredo Biological Marine Reserve (SC – Brazil) (*n* = 206)	The first maturation size was determined for females (L_50_ = 470 mm; r^2^ = 0.99). The relationship between the length and weight was W = 9 · 10^−6^ · TL^3.1149^ (r^2^ = 0.998; *n* = 246).	Andrade et al. (2003) [34] *Brazilian Archives of Biology and Technology*
Southern Brazil (SC) Babitonga Bay 2002–2004 (*n* = 193)	Collaborative approach The regression equation of the relationship between TL (mm) and TW (g) (TW = aTL^b^) was: TW = 4.4 × 10^− 5^ TL^2.8^, R^2^ = 0.97.	Gerhardinger et al., (2006) [36, 37]
Santa Catarina State 1998–1999	Habitat uses Water temperature	Machado et al. (2003) [59]
Itajái, SC	Food *Cronius ruber* (crab)	Daros (2005) [60] (undergraduate thesis)
Lab. Exp. (*n* = 27) Instituto Pesca, SP	Sexual inversion	Sanches (2009) (master thesis)
Patos Lagoon, South of Brazil (*n* = 108)	Otolith and gonads (growth and reproduction): “*K: 0.069 was lower than values reported for dusky grouper populations from the Mediterranean Sea (0.087) and southeast Africa (0.09)”* *“The current L*_*50*_ *estimate of 451.3 mm indicates that most individuals captured in this area are immature.”*	Seyboth et al. (2011) [61]
South of Brazil, Carpinteiro Bank (*n* = 201)	Age and growth 150–1160 mm Otoliths 1–40 years	Condini, Albuquerque & M. Garcia. *Fishery Bulletin.* 2014.
Southern coast	Mercury contamination in this species was correlated both with site locations and body sizes. Mature larger-body indi- viduals (N 650 mm and N 8 years old) exhibited the highest mercury concentrations (harmful to humans).	Condini et al. (2016) *Marine Pollution Bulletin*
Paraty and Copacabana, RJ	Grouper genetics Connected populations (Paraty and Ubatuba coasts) J Coast Zone Manag 2016, 19:2 These values suggest that within the geographic distribution of *E. marginatus* from Paraty to Rio de Janeiro, there are no subdivisions of the population. The effective population size (N_e_) was calculated for the only genetically differentiated group, K = 1, and resulted in 663 individuals between the Paraty (RJ) and Rio de Janeiro (RJ) populations.	Priolli et al. (2016) [35] (*Scientia Marina)*

The RMSD was calculated using the following equation:

$$ {D}_{RMS}=\sqrt[2]{\frac{\sum_{i=1}^n{\left({TW}_i-{ TW p}_i\right)}^2}{n}} $$

The Kullback-Leibler divergence was calculated by the following equation:

$$ {D}_{KL}\Big( TW\left\Vert TWp\Big)\kern0.5em =\kern0.5em \sum \limits_{i=0}^n\left({TW}_i\kern0.5em In\kern0.5em \left(\frac{TW_i}{{TW p}_i}\right)\right)\right. $$

where.

TW_i_ is the grouper weight from our sampling, and.

TWp_i_ is the grouper weight for the length of our sampling, as estimated by the equation.

Before calculating the Kullback-Leibler divergence, we normalized the data.

## Results

### Literature review: a summary of studies on dusky grouper

As previously mentioned, there are no marine reserves specifically for groupers in Brazil, although some reserves protect reefs and rocky areas where groups are expected to occur. Specific management procedures were very recently established (July–November 2018). This situation is completely different from what is found in the Mediterranean Sea. Therefore, we provide a brief description of the studies in Brazil concerning *Epinephelus marginatus*.

Studies on *E. marginatus* in Brazil have investigated the biology of the species, especially the development (e.g., growth, ontogeny, ecology) [21]. LEK has also been described in several studies (Table 1). LEK of the dusky grouper by small-scale fishers (especially those that use spearfishing and handlines) is available (Table 1). Such studies have addressed the habitat, reproduction and spawning calendar [57] of the dusky grouper. In other studies, the stomach contents were analyzed along with information on the local knowledge of fishers, who highlighted the importance of crabs in this fish’s diet [8, 19]. Crabs have specifically been shown to be an important component of the dusky grouper diet [63].

A review of information on groupers (and snappers) along the coast of Brazil was provided in Begossi et al. [19]. Folk taxonomy was also approached in a study including 38 fish species on the coast of Brazil [2].

Moreover, information on the studies of the weight-length relationship of the dusky grouper is provided in Table 1 [34, 36, 37], as well as information on the determination of its first maturation size in Brazil (females: L_50_ = 470 mm, r^2^ = 0.99) [34]. Most of this mentioned research was focused in south, i.e., Santa Catarina state [34, 36, 37, 61, 64] and Southeast Brazil, especially São Paulo state (Bertioga) [8] (Table 2). The weight-length curves are provided in Fig. 1, including those from our study at Copacabana, Rio de Janeiro (*n* = 221 individuals). For Copacabana specifically, we estimated TL_min_ = 38 cm and TL_max_ = 109 cm, but a 130 cm dusky grouper was previously found at Copacabana (Table 2).

**Table 2:** Estimation of the differences in the weight-length equations for the dusky grouper

Equation number	Equation	N	R^2^	TL_min_ –TL_max_, cm	Locality	Source
1	TW = 9*10^−6^*TL^3.1149^ (kg-cm)	246	0.9985	22–100.2	South of Santa Catarina State	(Andrade et al., 2003) [34]
2	TW = 8 * 10^−6^ * TL^3.2213^ or ln W = −11.76 + 3.221 ln L (kg-cm)	135	0.9828	25.1–79.6	Southeast Brazil (data from 1999 to 2000)	(Ximenes-Carvalho et al., 2012) [62]
3	TW = 0.0028TL^2^–0.143TL + 2.246 (kg-cm)	793	0.83	17–130	Copacabana Beach, Rio De Janeiro State	(Begossi et al., 2016)
4	TW = 4.4 * 10^− 5^ * TL^2.8^ (kg-cm)	173	0.97	30–100	Babitonga Bay and Sao Francisco do Sul Island, Southern Brazil	(Gerhardinger et al., 2006) [36]
5	TW = 0.0022TL^2^–0.888TL + 1.1079 (kg-cm)	183	0.9547	22–62	Southeastern Brazilian coast	(Begossi et al., 2012) [19]
6	TW = − 1173.00 + 5.23TL (g-mm)	22	0.84	23–48	Bertioga (coast of Sao Paulo)	(Begossi and Silvano, 2008) [8]
7	TW = − 3775.82 + 12.21TL (g-mm)	37	0.88	32–68	Copacabana (coast of Rio de Janeiro)	(Begossi and Silvano, 2008) [8]
8	TW = 0.0039TL^2^–0.2704TL + 5.9295	221	0.9612	38–109	Copacabana	Our study here

We compared the weight-length curves in Table 2 and Fig. 1.

Based on the Kullback-Leibler divergence, Eqs. 1, 3, 2, and 4 were the best approximations (i.e., D_KL_ ≈ 0.011 or 0.012), which were used in our polynomial approximating equation:

$$ \mathrm{TW}=0.0039\ {\mathrm{TL}}^2-0.2704\mathrm{TL}+5.9295 $$

According to the RMSD method, the best approximations were given by the following equations (in descending order): 3, 4, 5, and 1, which were acquired from Begossi et al. [2, 38] (Copacabana, Rio, SE coast), Gerhardinger et al. [36, 37] (Babitonga, southern coast), Begossi et al. [19] (Copacabana) and Andrade et al. [34] (southern coast).

The worst approximations of our data were given by linear fits from Ref. [6] (Table 2), which were in Begossi and Silvano [8] at Copacabana. These linear fits were obtained from samples of mostly small specimens, i.e., 25–40 and 30–50 cm. Thus, linear fits can be used for only grouper samples that are rather close in size and weight and with small parameter variations. This result is interesting because this early work was performed without the help of small-scale fishers; thus, we tended to collect species that were smaller than those found in the other samples from Copacabana. This difference occurred because many large individuals are sold quickly or separated from the landings to be sold to restaurants.

Other studies in Brazil (Table 1) focused on habitat use [59], food ingestion [60] and sexual inversion (experimental studies) [65]. These studies were concentrated in southern Brazil (Santa Catarina state). Other studies on age and growth included data on otoliths, as well as information on reproduction and mercury concentrations [21, 61, 66]. The genetics of the dusky grouper from the Rio de Janeiro state, was studied by Priolli et al. [35] using fin samples provided from the catches of local small-scale fishers. Fishing spots in Paraty were also identified [2, 8, 35, 38, 67, 58]. Other studies focused on habitat and water temperature [59], food items [60] and sexual inversion [65], as well as sexual transition [68]. Specific protocols of collaborative approaches between researchers and fishers were successfully employed by Gerhardinger et al. [36, 37] and Begossi et al. [2, 38].

The importance of the dusky grouper in terms of the food preferences and food security for the small-scale fishers of the Atlantic Forest coast has also been reported. For instance, the dusky grouper was mentioned by the fishers as a fish that is recommended for consumption during illnesses [56]; in addition, the dusky grouper is a preferred food item of the local population [38, 69]. Finally, considering the importance of dusky grouper in the market, another study [70], compared the color and texture of fresh and frozen dusky group filets.

Groupers are economically important, meaning that they have a market demand. For example, they are the most important fish sold in large food trade centers in Hong Kong and China ([23], p. ix).

Among the other studies on dusky grouper that have provided management suggestions, five focused on the biology of the dusky grouper (e.g., a review of its biology and otoliths, analysis of its growth coefficient, age, reproduction, sex change, and mercury concentrations, and population structure) [21, 61, 62, 64, 66, 71]. Two studies approached the mapping of habitats and the fishing spots used to catch dusky groupers [67, 72], and eight studies included more direct information on the ecology and conservation of dusky groupers, their fisheries, or the importance of MPAs to their conservation [2, 4, 6, 19, 35, 38, 48, 73].

Other aspects include examples of dusky grouper aquaculture in Brazil. More than ten years ago, Sanches [74] conducted very optimistic research on the aquaculture of groupers in Brazil. Currently, aquaculture centers for groupers are rare in Brazil. We visited Redemar Alevinos in February 2017 at Ilhabela, São Paulo state (private investment by C. Kerber). Larvae and juveniles of *E. marginatus* were observed in this area (SUPP. MAT.). Abroad, we visited centers in Dubrovnik and Split (Croatia), Heraklion (Crete, Greece) and Faro, Algarve, Portugal. In particular, a team in Faro successfully studied the aquaculture and recolonization of the dusky grouper (Dinis et al. [75]).

### The context of groupers in Latin America: the importance of local knowledge

The dusky grouper, *Epinephelus marginatus* does not occur in the northern part of Latin America, including the Caribbean area, but other species of *Epinephelus* occur, including *E. morio*.

In Latin America, other countries than Brazil seem to be midway between the management gaps found in Brazil and the more structured Mediterranean MPAs. There are important examples concerning initiatives to manage groupers in Latin America including the use local ecological knowledge in these initiatives.

At Yucatan, Mexico, *Epinephelus morio* represents 30% of the state total fish catch; as groupers have probably declined, *E. morio* has been substituted by *Mycteroperca bonaci* in fish catches [76]. Galindo-Cortez et al. [77] showed that the groupers *E. morio, E adscensionis, E. drummondhayi, E. guttatus*, *E. itajara,* and *E. striatus* are important species in the finfish fisheries in the southern gulf of Mexico and in the Caribbean sea; some of these species has been managed through closed fishing seasons (Campeche Bank) and minimum length (*E. morio*).

Fulton et al. [78] stressed that in spite the participation of fishers (through local ecological knowledge) supporting scientists and managers, fishers are often excluded from decision-making processes: for that reason, community-based monitoring models were build up in three marine marine ecosystems in Mexico, with the participation of 400 artisanal fishers. In another study in Yucatan, Mexico, Fulton et al. [79] showed the importance of complementary approaches to science, by including traditional ecological knowledge and citizen science in detecting fish spawning aggregations of groupers and snappers, such as *E. striatus*. Still at Yucatán, Mexico, a study by Aguilar-Perera et al. [80] approaching especially *Epinephelus itajara*, but adding information about *E. morio*, emphasized the importance of local knowledge in reconstructing historical records. Snappers and groupers account for 93% of Gulf Mexico fisheries: these are data-poor fisheries which have been counting upon local expert/local knowledge, especially concerning spawning ground aggregations [81].

Other countries from Latin America have been using local ecological knowledge to manage fisheries within ecosystem-based approaches. In Panama, red snapper and grouper are examples [82]; in Colombia, historical changes were detected using local ecological knowledge for “mero” groupers and “pargo” snappers [83]; in Porto Rico, García-Quijano and Pizzini [84] approached several ecological attributes, through local ecological knowledge, for several species, including *Epinephelus guttatus* and *E. mystacinus*.

### Management and data-poor fisheries

Studies that compared population densities inside and outside MPAs have shown that the densities of groupers (Epinephelidae) and related reef fish (Serranidae) have increased within MPAs, which has also helped to maintain the ecological services provided by these large reef predators [85–88]. MPAs also have the potential to maintain the stocks of groupers and other reef fish in adjacent areas through the spillover of adults or dispersal of larvae [89]. However, MPAs may not always be effective or the best way to manage fish and fisheries due to the lack of scientific support (ecological data), increased conflicts with local fishers and enhanced fishing pressure in neighboring unprotected areas, among other limitations [90, 91]. Other limitations to the proper evaluation of the effectiveness of MPAs include the lack of a before-and-after control and impact (BACI) sampling design in many studies addressing MPA effects, which usually lack data from before the protected area was established [47]. More specifically, in terms of the conservation of the dusky grouper, our review (SUPPLEM. MAT) indicated that MPAs have been more effective in the Mediterranean than along the Brazilian coast. For example, a) many Mediterranean MPAs are located around or adjacent to islands, since isolation increases the consequences of nursery habitat deficiency; b) the network created by the many small reserves along the Mediterranean generates positive outcomes for connectivity and conservation; and c) zonation is almost always practiced in the Mediterranean [5].

Finally, for data-poor fisheries, such as small-scale Brazilian fisheries, it is especially important to consider LEK during the acquisition of additional information on a species. In fact, among the 65 species along the coast of Brazil that were identified by fishers as being the ‘most consumed’, 54% have an unknown status [69]. Based on data from Latin American fisheries, FAO (FAO Technical Paper [92]) has emphasized the importance of fishers’ knowledge in the ecosystem-based approach to fisheries, in special in developing countries where data-poor fisheries are common. Therefore, in South American areas, especially is Brazil where fishery statistics (including coastal areas) are very scarce and little is known about most species, LEK is very important to subsidize management.

### Results from fieldwork (landings, interviews and markets)

#### Dusky groupers in the small-scale fisheries of Copacabana, São Sebastião and Santos

**Fig. 2:**
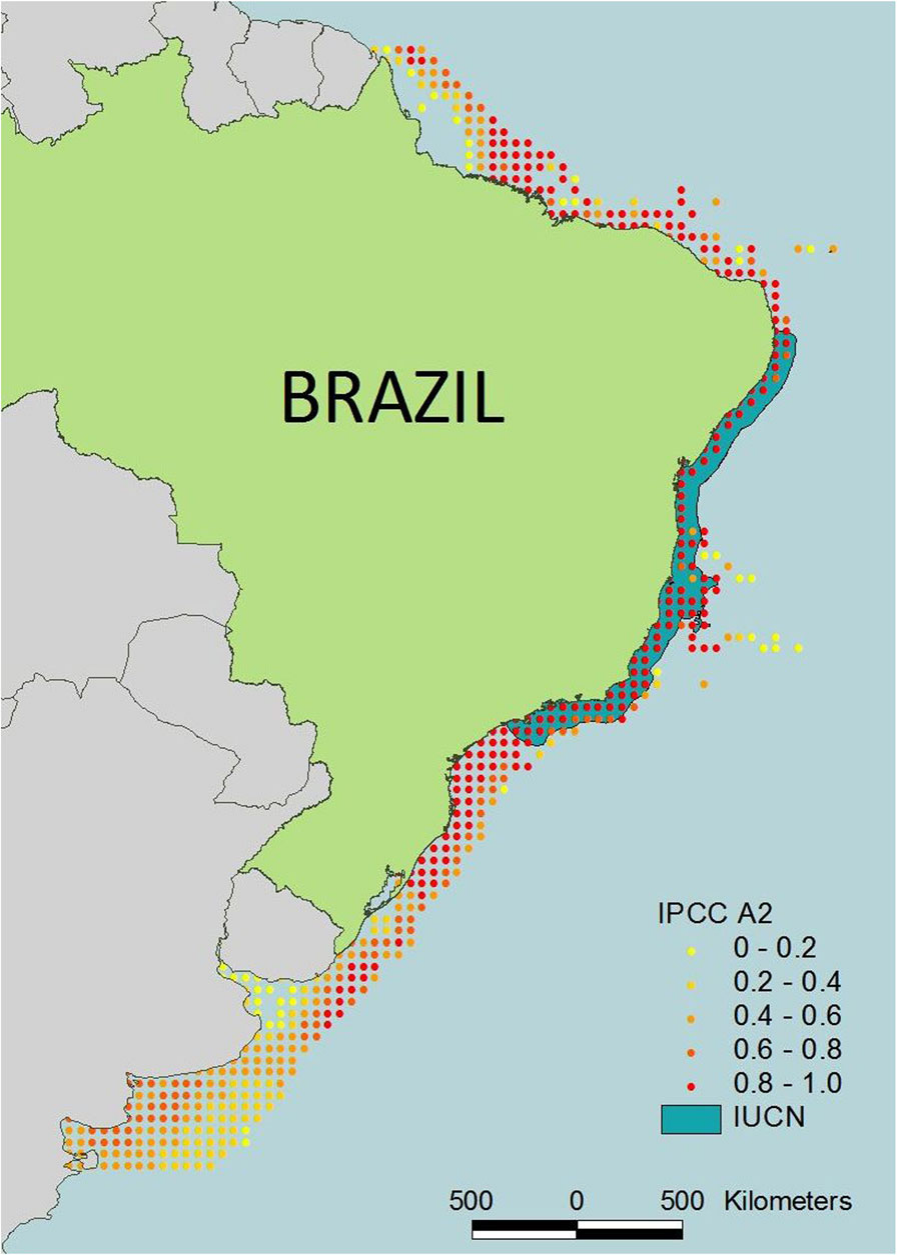
Distribution of Epinephelus marginatus (Dusky grouper) based on the IPCC A2 emissions scenario [93] and The IUCN Red List of Threatened Species 2004 [Cornish, A. & Harmelin-Vivien, M. (Grouper & Wrasse Specialist Group) 2004. Epinephelus marginatus.: e.T7859A12857009. World wide web electronic publication, 10.2305/IUCN.UK.2004.RLTS.T7859A12857009.en. Accessed 8 Oct. 2018]

The dusky grouper distribution is shown in Fig. 2. As indicated by this figure, we should expect to find this species in Northeast Brazil.

Several sites were visited to identify dusky groupers (*Epinephelus marginatus*). Table 3 shows the different sites that were visited in this study, from the northeastern state of Rio Grande do Norte to the southernmost state of Rio Grande do Sul (Table 3).

**Table 3:** Locations where *Epinephelus marginatus* and *E. morio* were observed

States, Sites and Markets	Location and number of markets visited	Species *Epinephelus*
Rio Grande do Norte^c^	(I) Natal (Ponta Negra): 2	
(7)	Natal (Redinha): 1	
	Pirangí do Sul: 3	
	Tibaú Sul: 1	
Paraíba^c^ (1)	João Pessoa (Tambáu): 1	*E. morio*
Pernambuco^c^ (2)	(I) Cabo de S. Agostinho (Gaibu e Calhetas): 2	
Alagoas^c^ (11)	(I) Maceió (Praia do Francês): 1	
	Barra de S. Miguel: 1	
	Jequiá da Praia: 1	
	Lagoa Azeda: 4	
	Pontal do Coruripe: 2	
	Piabucú: 2	
Sergipe^c^ (1)	Central market of Aracaju: 1	
Bahia^c^ (11)	(I) Praia do Forte (Mata S. João): 3	*E. morio* (Praia do Forte, Arembepe and Salvador)
	Santo Antonio: 1	
	Imbassaí: 1	
	(I)Arembepe: 3	
	(I) Salvador (Itapuã): 3	
Bahia^b^ (31)	(I) Porto do Sauípe: 1	*E. morio* in Ilhéus, Valença and Salvador. Catches from: Alcobaça, Canavieira, Porto Seguro, Itapuã, Belém, and Fortaleza.
	Praia do Forte: 2	
	Arembepe: 2	
	Salvador: 7	
	(I)Ilhéus: 2	
	(I)Acuípe: 2	
	(I)Itacaré: 3	
	(I) Pedras do Una: 1	
	Camamu: 2	
	Ituberá: 2	
	Valença: 5	
	Itaparica: 2	
Bahia^d^	Prado	*E. morio*
Rio de Janeiro^a^	Copacabana	*E. marginatus*
São Paulo^a^	(I)S. Sebastião	*E. marginatus*
	(I)Santos	
	(I)Bertioga	
Santa Catarina^e^ (Florianópolis) (8)	(I) Pântano do Sul^e^: 1	*E. marginatus*
	Downtown, center markets^e^: 2	
	Armação^e^: 1	
	Campeche^e^: 2	
Rio Grande do Sul^f^ (2)	Rio Grande: 2	*E. marginatus*
	Torres: 2	

^a^Landings were followed systematically at Copacabana, S. Sebastião and Santos

^b^Fieldwork conducted in November 2016 at Bahia

^c^Fieldwork conducted in March 2018: Rio Grande do Norte to Bahia (Praia do Forte and Arembepe)

^d^Fieldwork at Prado, Bahia, March and April, 2017

^e^Fieldwork at Santa Catarina, Florianópolis, Pântano do Sul, March 2017

^f^Fieldwork Rio Grande do Sul, December 2016 and March 2017

**Fig. 3:**
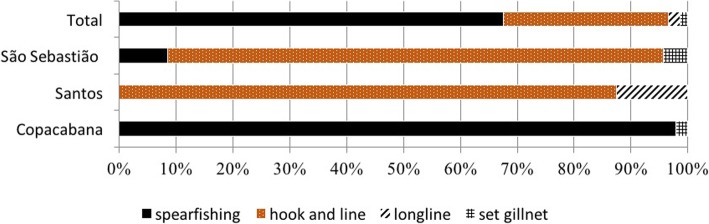
Gear used (landings) to catch dusky grouper (*E. marginatus*) in Southeast Brazil from São Sebastião (*n* = 47), Santos (*n* = 80) (Sao Paulo state) and Rio de Janeiro (Copacabana) (*n* = 291)

**Fig. 4:**
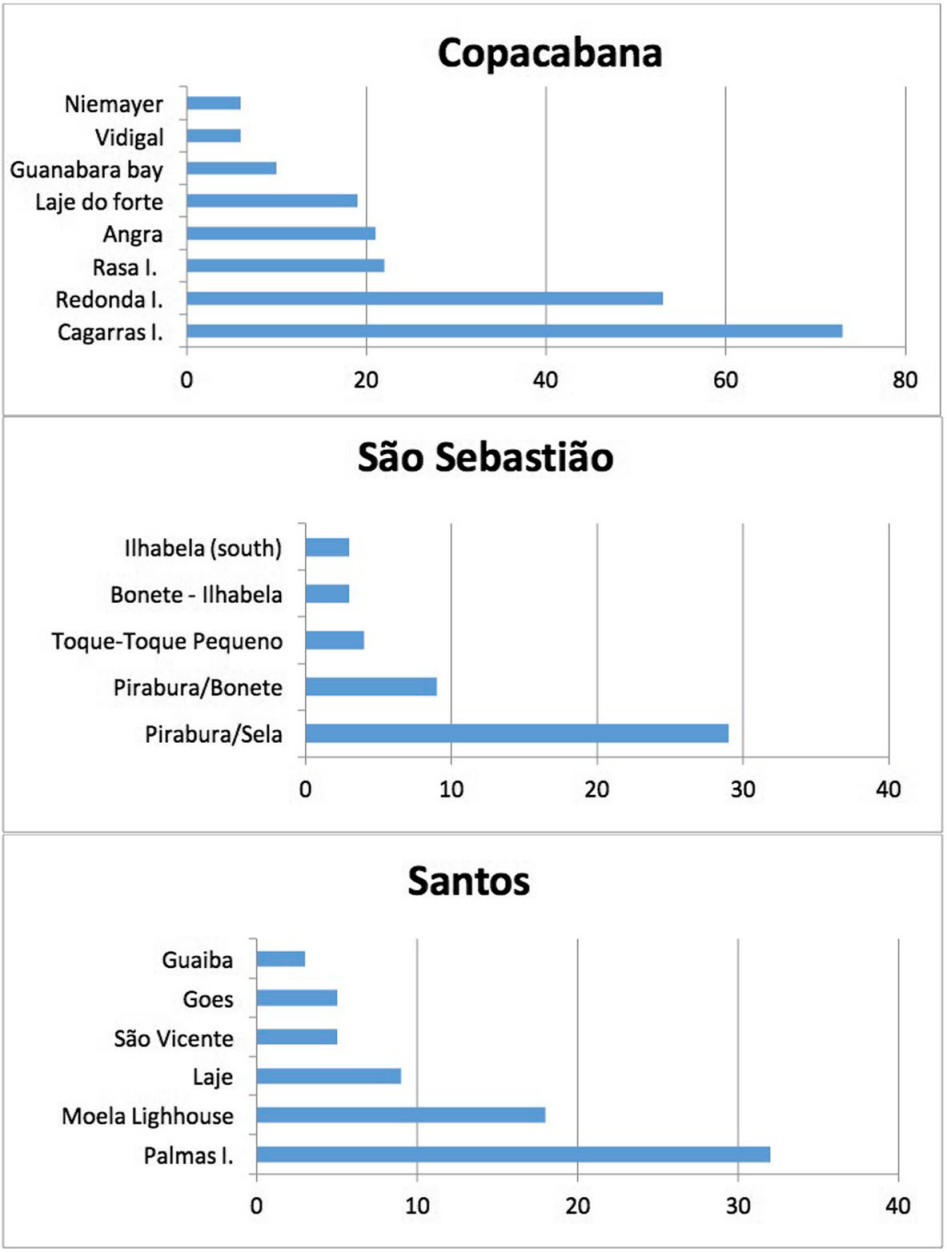
Number of fishing trips per fishing spot at Copacabana (RJ), Santos and São Sebastião (SP). See Table 4 for additional information (landings) (the correct name is Niemeyer)

**Fig. 5:**
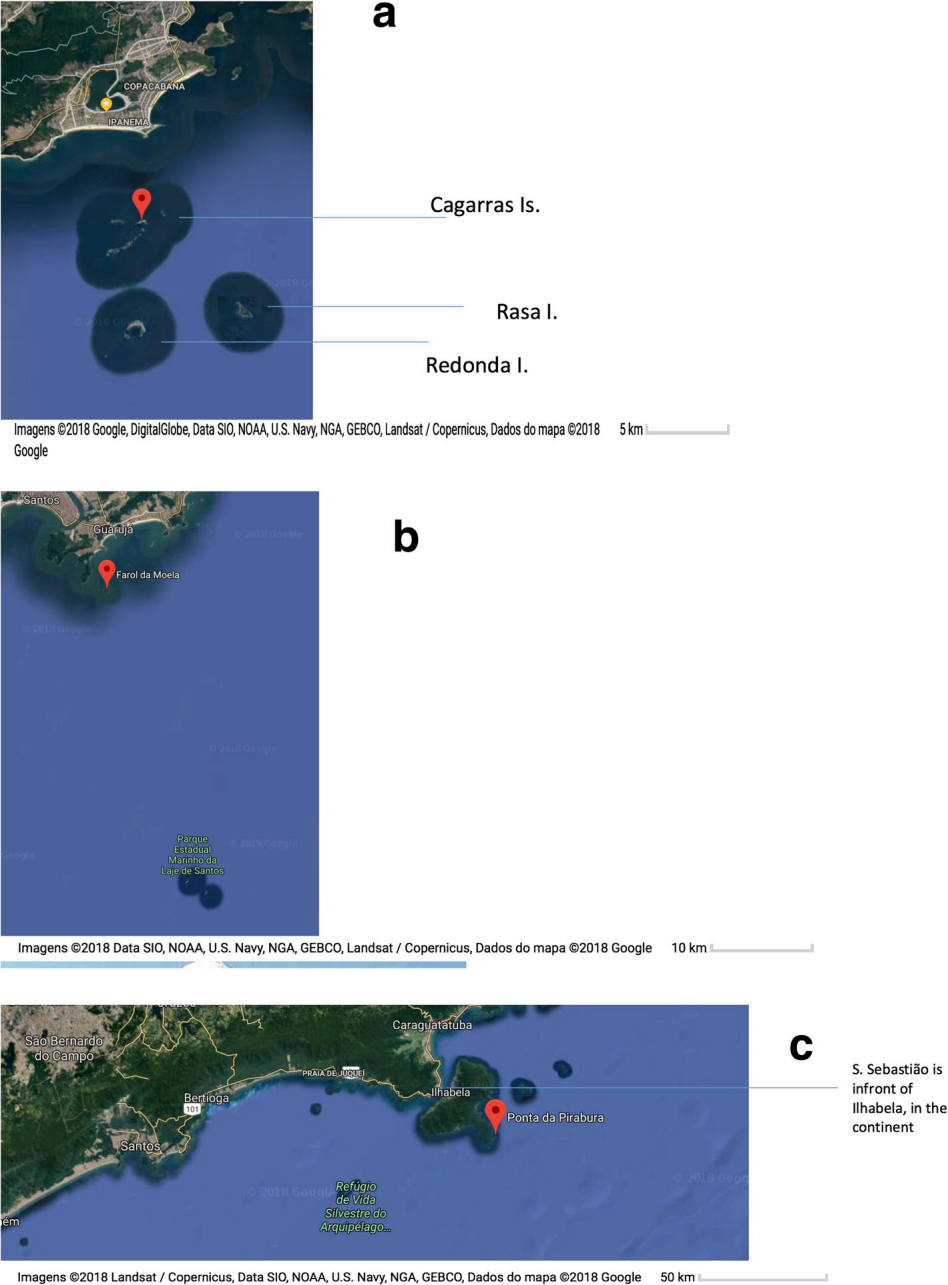
**a**. Main spots used by fishers from Copacabana (Posto 6), Rio de Janeiro to catch dusky groupers (see Table 4, landings) (islands Cagarras, Rasa and Redonda). **b**. Main spots used by fishers from Santos (SP) (Farol da Moela) to catch dusky groupers (see Table 4). **c**. Main fishing spots from the landings from São Sebastião , such as in the continent, in front at Ilhabela island (Table 4)

Landings were systematically followed at the ‘Colonia de Pescadores do Posto 6, Z-13’, Copacabana (RJ), São Sebastião and Santos (SP). The ‘Posto 6, Copacabana’ was created in 1923 and is one of the oldest fisheries associations located in the heart of Rio de Janeiro (Table 3). In this area, groupers have been a target and are considered highly appreciated fish with high market prices [94]. Fishing at Copacabana Beach is performed using small-scale motored canoes or boats using nets, hooks and lines, and by diving (i.e., spearfishing). In particular, the dusky grouper is caught by spearfishing (Fig. 3). Recently, spearfishing through free diving has become important, especially among young fishers. Data from the observations of dusky groupers are shown in Table 4. As shown in earlier studies [2, 8, 19, 38], dusky groupers continue to be caught around the Cagarras Islands, which is an archipelago that is relatively close to the Copacabana and Ipanema beaches. Two islands, Redonda and Rasa, are also commonly used by fishers from Copacabana to catch groupers (Figs. 4 and 5).

**Table 4:** Number of groupers per fishing spot and study site (Copacabana, S. Sebastião and Santos). Landings were followed for Santos (August, 2016-March, 2018); S. Sebastião (June 2016-November, 2016) and Copacabana (April 2016-November, 2016)

Site/Trips	Fishing spot	Number of groupers
Copacabana Total = 222	Cagarras	73
	Redonda	53
	Rasa	22
	Angra	21
	Laje do forte	19
	Baia Guanabara	10
	Costão do Vidigal	6
	Costão do Niemeyer	6
	Posto 6	1
	Maricá	1
	Laje da cagarra	1
	Cabo frio	1
	Arpoador	1
	Macaé	1
	Sem dados	6
Santos Total = 80	Ilha das palmas	32
	Farol da moela	18
	Laje	9
	Goes	5
	S. Vicente	5
	Ponta Grossa	4
	Guaíba	3
	Others (Mandubo/Saugana)	3
Local São Sebastião Total = 47		
	Pirabura/Sela	29
	Pirabura/Bonete	9
	Toque-Toque Pequeno	4
	Bonete - Ilhabela	3
	Sul da Ilhabela	3
	Ponta do Boi – Ilhabela	1

#### São Sebastião

São Sebastião is a small municipality on the northern coast of São Paulo state. Bordered by the Atlantic Rain Forest, São Sebastião includes 34 beaches, most of which are inhabited by artisanal fishing communities, such as Enseada, São Francisco, Porto Grande, Araçá Bay, Toque-Toque Pequeno, Boiçucanga, and Barra do Sahy [95]. São Francisco Beach is also a traditional fish landing point used by fishers from nearby islands (e.g., Ilhabela and Búzios).

The fishers from these comunities practice artisanal coastal fishing, using paddled canoes, motored canoes or small boats, trawling nets, gillnets and hooks and lines, and some young fishers practice spearfishing. Dusky grouper is mainly caught with hook and line gear (Fig. 3). The main spot used to catch dusky groupers is Ilhabela Island, one of the largest islands off the coast of Brazil (Figs. 4 and 5; Table 3).

Santos is a large coastal city located on the southern coast of São Paulo state. Our research was performed at the Mercado do Peixe da Ponta da Praia, where we collected data from the grouper landings of one fisher (Table 4), who also worked at the market. Dusky groupers are also mainly caught with hook and line gear in this area (Fig. 3), especially at sites with rocky shores, outcrops and islands (e.g., Palmas Island, Moela lighthouse and Laje) (Figs. 4 and 5; Table 3).

The small-scale fisheries of this study do not have communal fishing: catches come from hook and line, set gillnets and spearfishing, in small crews of 1–5 fishers. Small crews made up of relatives or friends are common and kinship plays important role in catches and in territorial rights [10].

#### Landings of dusky groupers

At Copacabana, landings data also revealed that the groupers that were caught ranged in size from 38 to 109 cm and the weight range was 1–24 kg, with an average weight of 3.84 kg (std = 3.14, *n* = 222). The largest grouper was caught at Cagarras. At São Sebastião, the size range was 39–106 cm, and the weight range was 2–8 kg, with an average weight of 2.77 kg (std = 1.37; *n* = 47). At Santos, the size range was 26–120 cm, and the weight range was 0.36–15 kg, with an average weight of 2.72 kg (std = 2.76; *n* = 80).

We also sampled 44 landings (from June 28, 2016, to July 7, 2016) from a fishing club adjacent to the Copacabana Fishery Association, where sport fishers bring catches from Cabo Frio (NE of the state of Rio de Janeiro). The average size of these groupers was 5.07 kg (sd = 4.97) (range: 40–68 cm and 38–24 kg).

The main fishing spots used by the fishers who landed at these areas were the rocky islands relatively close to shore (Fig. 5). From the 847.46 kg of groupers (*n* = 222 trips) landed at Copacabana, 292.32 kg (i.e., 34%) came from Cagarras, 184.12 (i.e., 22%) from Angra dos Reis, 109.01 kg (i.e., 13%) from Redonda Island, and 89.49 (i.e., 11%) from Rasa Island. At São Sebastião (*n* = 47 trips), Pirabura was the main and most productive spot (a few catches from Bonete were included because some landings data were combined): 141 kg of 196 kg (75%). The Santos data (119 kg of groupers from 34 trips) included the Laje ground, with 63.98 kg (i.e., 54%), followed by Moela lighthouse with 21.44 kg (i.e., 18%), and Palmas Island with 16.04 kg (i.e., 13%).

Using the landing data and macroscopic observations of the gonads [2, 38], we observed only two (110 and 400 ml) mature gonads in Copacabana, both of which were weighed (vol.) in November 2018. No mature gonads were observed at São Sebastião, while five were registered in Santos (two in October 2016: 48 and 50 ml, two in November 2016: 300 and 350 ml, and one in January 2017: 150 ml).

#### Larvae collection and fish observation

We found copepods, cladocerans, shrimp and fish larvae were found (no dusky grouper larvae were observed) in the trials performed at Cagarras I., Copacabana.

**Fig. 6:**
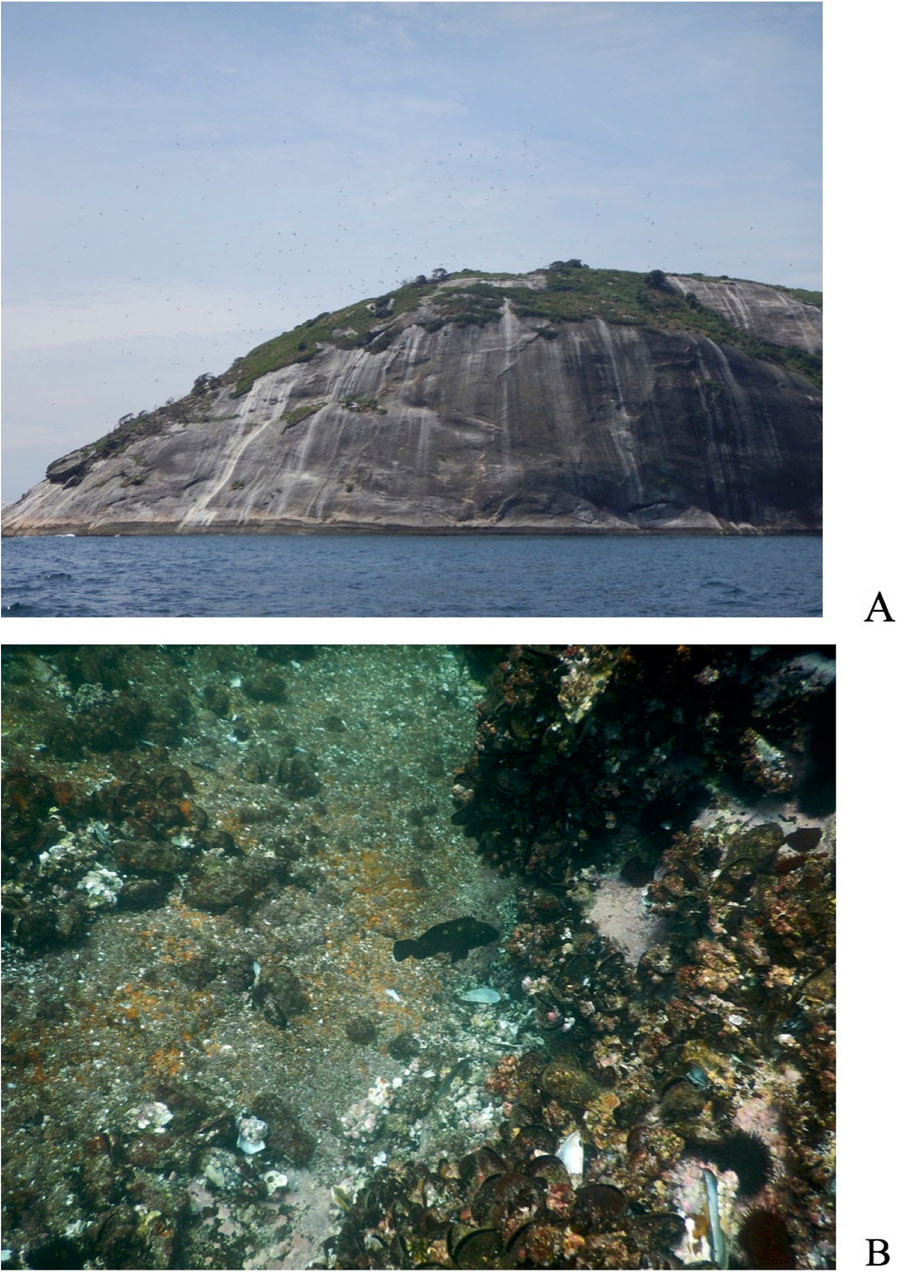
**a** Main island of the Cagarras Archipelago where diving was performed (Photo: Renato Silvano). **b** Dusky grouper (*Epinephelus marginatus*) with a length of approximately 35 cm observed during free diving at Cagarras Island (Rio de Janeiro) (Photo: Renato Silvano)

Diving was also performed at Cagarras Island, where only one juvenile *E. marginatus* was observed; however, other grouper species were registered (Table 5 and Fig. 6).

**Table 5:** Data from diving performed at Cagarras Archipelago, Copacabana, RJ (January, 2018), with the number of fish observed in each diving step (N)

Site	Date	Depth (m)	Hour Start	Hora End	Length (min)	Species	N	Size (cm)	Visibility (m)
Comprida I.	09/01	12 a 15	12:06	12:57	51	*Mycteroperca acutirostris*	1	40	6
Rasa I.	10/01	10 a 12	09:28	10:23	45		0		8
Ilha da Praça 11 I.	10/01	8 a 11	11:30	12:10	40	*Epinephelus* sp.	1	20	6
Matias I.	11/01	2 a 5	09:20	10:20	60	*Epinephelus marginatus*	1	35	5
Comprida I.	11/01	2 a 5	10:45	11:25	40	*Mycteroperca acutirostris*	1	45	5

#### Folk knowledge on the coast of Brazil: *Epinephelus marginatus* (SE and S) and *Epinephelus morio* (NE)

**Fig. 7:**
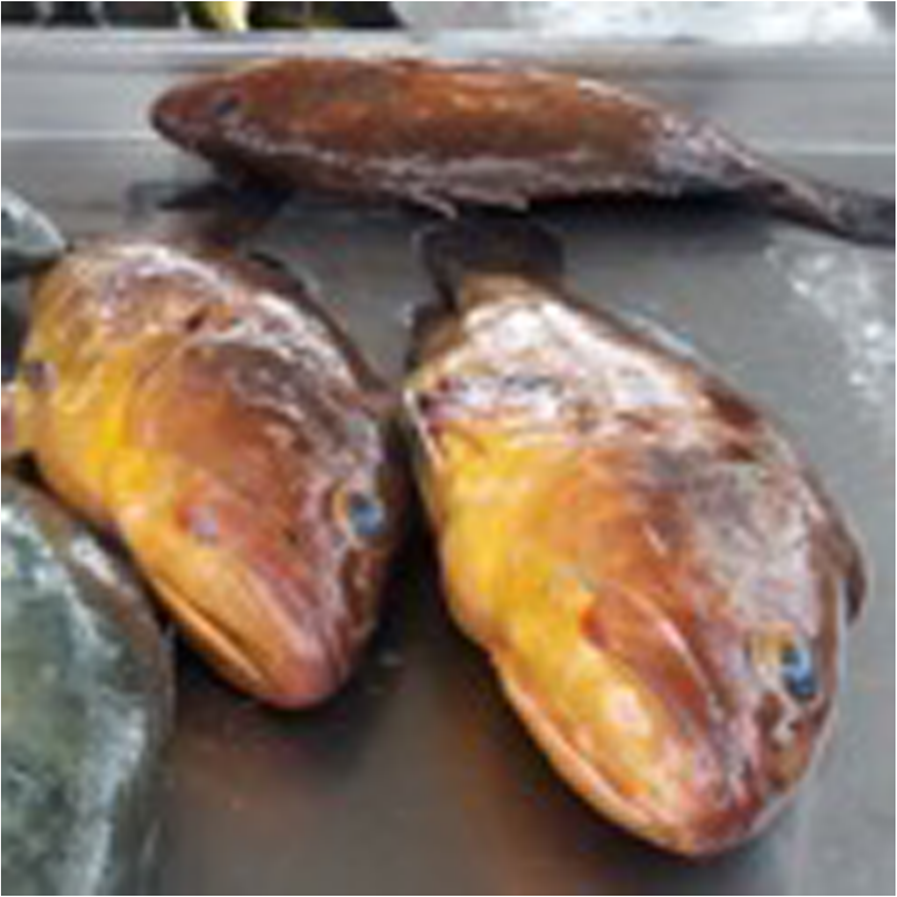
Dusky grouper (*Epinephelus marginatus*) shown at Posto 6 to be sold by fishers. These pictures were shown to fishers during interviews

Our results included trips and interviews with 81 fishers from Rio Grande do Norte, northeastern Brazil to Santa Catarina, southern Brazil. We conducted informal interviews or chats (*n* = 10) along the northern coast of Brazil in Natal (Rio Grande do Norte state, RN), Cabo de Santo Agostinho (Pernambuco state, PE) and Maceió (Alagoas state, AL) (Table 3). The interviews were discontinued in this part of Brazil (RN, PE and AL) because the fishers did not recognize a picture of *E. marginatus* and many did not consider it a grouper (Fig. 7). Our total of fishers is 71 in Additional file 1: Table S1, since the informal interviews were not included in this Table. Therefore, the results on local knowledge came from 71 fishers from the northern part of Bahia state (Porto do Sauípe) to the Santa Catarina coast (Florianópolis) (Additional file 1: Table S1). In Northeast Brazil, some of this information could have been about *E. morio* because fishers considered it rare or ‘disappeared’.

The fishers interviewed had a mean age of 54 years, with 32 years of fishing experience and 40 years of residence. Considering the different research areas, we approached 25 fishers from Bahia (NE), 11 from Rio de Janeiro, 25 from São Paulo (SE), and 10 from Santa Catarina (S) (Additional file 1: Table S1). The mean ages varied from 49 (Rio) to 58 (Bahia) years old; the mean fishing years varied from 29 (SP) to 39 years (SP), and the mean residence time was from 38 to 43 years (both in Bahia). Therefore, we interviewed skilled fishers with knowledge of their living areas and small-scale fisheries (Additional file 1: Table S2).

We obtained 66 responses to the question regarding the recognition of a picture of a dusky grouper (‘Do you know this fish?’ and ‘What is its name?’). From these responses, 92% called it ‘garoupa’ and 10% called it ‘garoupa verdadeira’ (‘real grouper’) (i.e., mostly overlapped responses). The fishers from Bahia commonly complemented their answers by stating that ‘garoupa’ used to be seen in the area, but the species is currently rare or has disappeared (six fishers from Bahia emphasized this point during the chats). The fishers from Bahia and from other regions of NE Brazil did not recognize the picture: at Ponta Negra (RN), for example, they named it sirigado (*Mycteroperca bonaci* or *Mycteroperca* spp.). We discontinued the interviews from Porto do Sauípe (Bahia) to Ponta Negra (Rio Grande do Norte) because it was clear that the fishers did not recognize the picture of *Epinephelus marginatus* shown to them. Thus, in that area, we had informal chats with the fishers and understood that they did not see the species in that area (which was different, for example, from Bahia at Praia do Forte and other areas further south). However, it is important to emphasize that because we did not observe *Epinephelus marginatus* from Bahia northward, we should assume that the responses from Bahia and further north could refer to (or also to) *Epinephelus morio.* Fishers from Bahia commented that *E. marginatus* was seen before, but it was rare.

The habitat of the dusky grouper was well known to the fishers (*n* = 71), as most (*n* = 92 citations) believed that the species is found in rocky shores, islands and sea slabs. In Bahia, a few fishers (*n* = 6) mentioned that the dusky grouper is caught between 50 and 126 fathoms (i.e., 28–70 m). Again, rocky shores and islands were mentioned as the primary spots used to catch groupers.

The diet of the dusky grouper was also described (*n* = 70) as fish (*n* = 30), especially sardines (*n* = 24), as well as other species, such as crabs (*n* = 23), squid (*n* = 14), octopus (*n* = 12) and shrimp (*n* = 11). Many fishers (*n* = 26) also said the dusky grouper eats ‘anything’, including ‘rotten’ food items.

The most frequently gear (*n* = 71 interviews) was hook and line (*n* = 66), followed by spearfishing gear (free diving) (*n* = 11), longlines (*n* = 9) and set gillnets (*n* = 7). In contrast, all fishers from Bahia (NE), São Paulo (SE), and Santa Catarina (S) catch dusky groupers using hook and line gear, and most fishers (73%) in Copacabana spearfish for the species. A few fishers on the coast of São Paulo (12%) spearfish for the species as well.

**Fig. 8:**
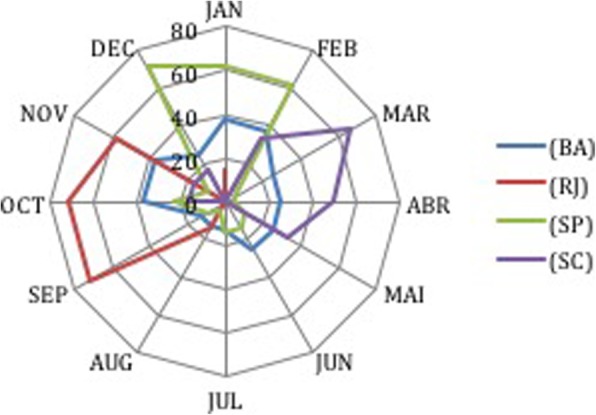
Monthly distribution of the time of gonad maturation in different locales (%) (interviews, *n* = 42)

**Fig. 9:**
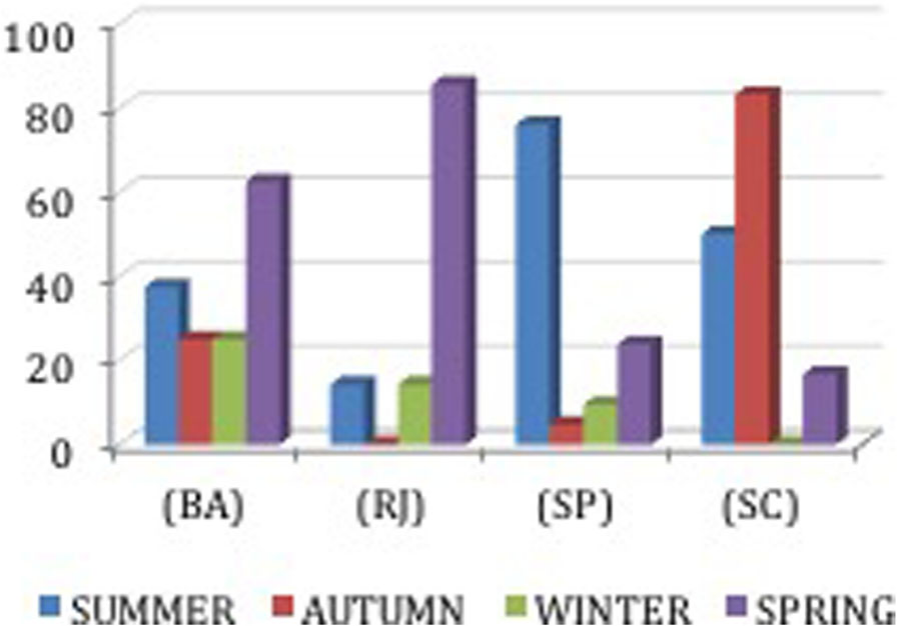
Seasonal distribution of the time of gonad maturation in different locales (%) (interviews, *n* = 42)

The period of reproduction for the dusky groupers (i.e., when gonads mature) was known by a few fishers from Bahia (8 out of 25); however, more fishers were familiar with this information in the areas further south (Rio, 7 of 11; São Paulo 21 of 25 and all from Santa Catarina). The reproduction was identified as around September (beginning of spring) to March (end of summer) (Fig. 8). Fishers from Santa Catarina, however, also mentioned that reproduction occurred in April and May (autumn). From the north to the south of Brazil, we observed the following reproduction periods (from fishers’ interviews): coast of Bahia (NE) and Rio de Janeiro (SE), mostly spring; coast of São Paulo, summer; and coast of Santa Catarina, summer and autumn (Fig. 9).

Fishers showed little knowledge of aggregations and larvae. A few fishers (28 of 71) replied that groupers aggregate; of these, 10 fishers mentioned that this aggregation was for reproduction, while six mentioned it was a feeding aggregation. Most fishers (82%) never saw grouper larvae, and 6% did not know (*n* = 71). However, only 11% said they saw larvae and one saw larvae after spawning.

Historically, the previous individual experiences of fishers with groupers were also considered during interviews. Although not all of the fishers responded, 53 replied, and the average size of the grouper was reported to be 73 cm (30_min_ and 150_max_); at São Paulo, the largest size was 150 cm. The average largest size observed by fishers (*n* = 61) was 100 cm (15_min_ to 200_max_). A maximum size of 200 cm was reported south of the Bahia, Rio de Janeiro and São Paulo coasts. The year of the reported observation was 20 years ago or longer for 12 fishers (Bahia State: 1 fisher, Rio de Janeiro: 4, São Paulo: 4 and Santa Catarina: 3). Six fishers reported observations from 10 years ago or longer (Bahia: 3, São Paulo: 1 and Santa Catarina: 1). Many groupers were mentioned in catches, especially by fishers from Santa Catarina (50, 30 and 20 groupers), São Paulo (50 and 30) and Rio de Janeiro (20).

#### Consumption and conservation

We visited 29 restaurants in the coastal areas of the states of Bahia (Arembepe, Porto do Sauípe and Praia do Forte), Rio de Janeiro (Copacabana), São Paulo (São Sebastião, Caraguatuba, Santos, Guarujá and Praia Grande), and Santa Catarina (Pântano do Sul) (Additional file 1: Table S3). An average of 347 kg/month of fish was purchased from fishers or fisheries to serve consumers. A few of the restaurants (7) served garoupa (grouper), while others served badejo (*Mycteroperca* spp.) (6). In Bahia, the grouper species was *Epinephelus morio*. Restaurants usually served groupers as ‘posta’ (transversal cut steak) or ‘moqueca’ (fish stew in a spicy sauce). Frozen filets were found in markets from Florianópolis (Additional file 1: Table S3).

**Fig. 10:**
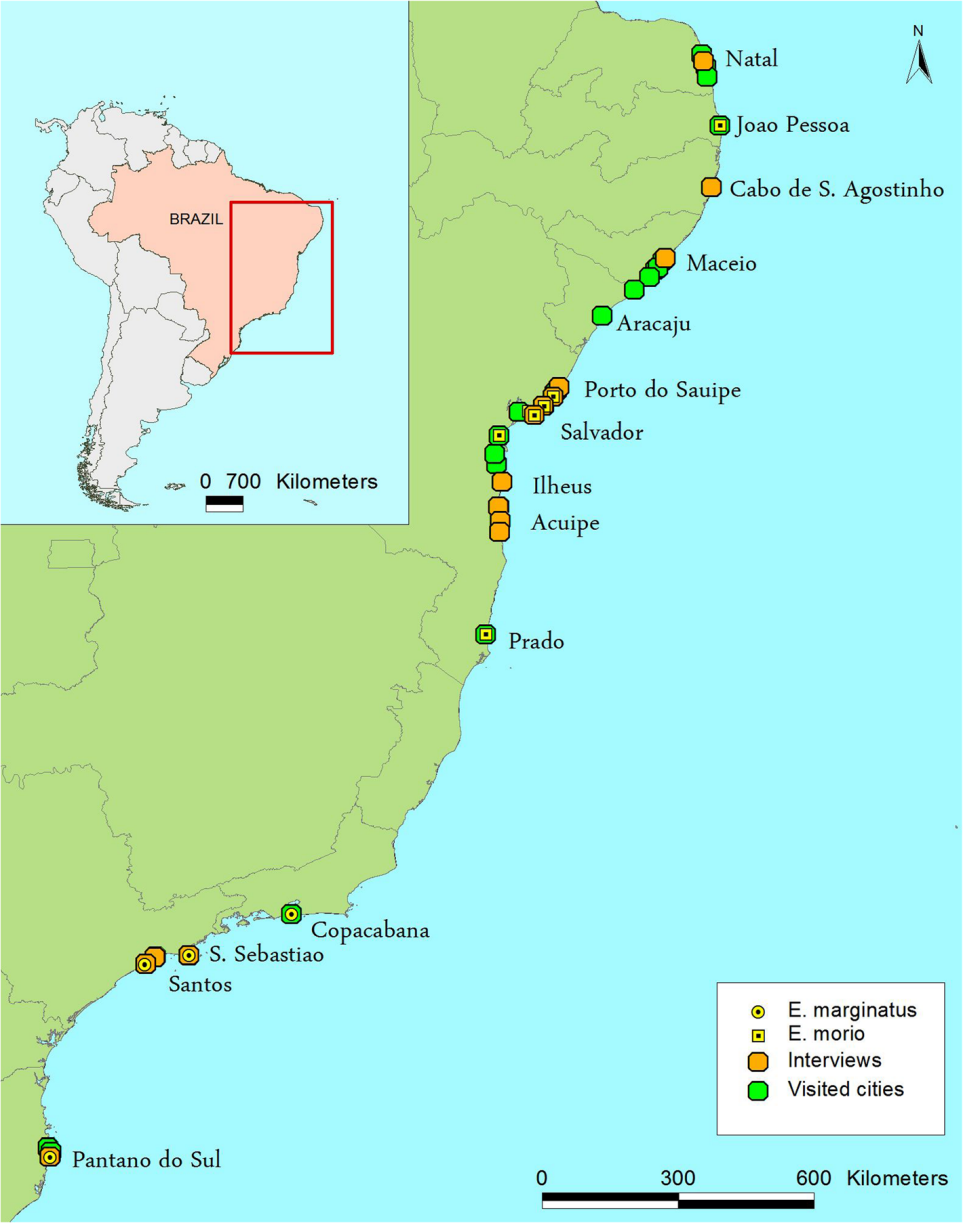
Map of the areas visited in Brazil

We could register the ex-vessel prices of fish sold to consumers or restaurants in only Copacabana. The prices (in Brazilian Real) are shown in Fig. 10a and b. When we began to follow the prices (October 12, 2016), the exchange rate was R$3.20; when we completed the sampling of prices (November 7, 2017), the dollar exchange rate was R$3.27. The prices were highest in November (both years) and June of 2017. Groupers were sold to consumers and, in particular, two different Japanese restaurants. The average price during this period was R$35.00 reais per kg (sd = 2.84) (SUPP. MAT.).

We also visited 74 markets from Natal (Rio Grande do Norte) to Florianópolis (Santa Catarina) (Table 3). *Epinephelus marginatus* was found in the markets from Rio de Janeiro to the southern part of Brazil. *Epinephelus morio* was found from Bahia to the northern states (Table 3 and Fig. 10). We observed *E. morio* at Ilhéus, Valença and Salvador and other sites south of Bahia (Fig. 10). The northernmost finding of the dusky grouper was from landings in Cabo Frio (RJ). Despite having indicators from Froese and Pauly [22] regarding the distribution of dusky grouper in the south of Bahia, we and the fishers did not observe this species (except for rare and past occurrences from south of Bahia up to Salvador).

#### Conservation of *Epinephelus marginatus* and *E. morio*

Because both species of groupers that were considered in this study are highly prized in the market and targeted by small-scale fisheries, they should be adequately managed, which includes measures to avoid or minimize conflicts with fishers (see Additional file 1: Table S4 for a summary of suggestions for Brazil and the Mediterranean). Examples of management approaches that could be applied to groupers are the establishment of a minimum capture size, the implementation of fishing bans during spawning seasons, the definition of coastal zone uses, participation by small-scale fishers in the decision-making processes, ecological and economic mechanisms such as payments for environmental services, improvements to MPAs, the planning of MPAs according to larvae distribution and population connectivity, and the mapping of fishing spots and area zonation.

## Discussion

Small-scale fishers have a special focus on this species, as it is a highly appreciated fish with good market prices (for more on the dilemma of consumption and selling, see Begossi and Richerson [96] (Additional file 1: Table S5).

Should dusky grouper be considered a key cultural species? Salience, cultural species or key cultural species are categories that express cultural importance. Ecological salience was suggested by Hunn [97], by considering that a salient organism (abundant, venomous, beautiful, among other attributes) is more easily perceived by individuals; this perception, however is influenced culturally, since different individuals have different chances to perceive the same organisms (fishers perceive easily aquatic organisms, for example). Later, Garibaldi and Turner [98] defined key cultural species as the salient species that are culturally important for a community, such as showing multiplicity of uses, nomenclature, symbolism, memory, difficulty of replacement by other native species, and provision of resource. In 2009, Platten and Henfrey [99] complemented this concept, by adding that a cultural keystone species has role in the maintenance of the complexity of the social-ecological system: their example of cassava (*Manihot esculenta*) shows the central role of this cultivar within the community. From these definitions, dusky grouper is a key cultural species, since it pertains to cultural domains of food taboos and local medicine in many coastal communities in Brazil [100]; it is a noble species, economically important with probably no ‘replacement’ by others, besides being colorful and reaching reasonable sizes. Different sources show groupers reaching in Brazil the size of approximately 110 cm (Fig. 1). In Hunn [97] terms the adult of dusky grouper is ecologically salient. Contrastingly, the larvae of grouper are very small to be observed by fishers; thus, fishers are not aware about when and where they occur.

We observed that dusky grouper is sold to consumers at a relatively high price compared with other relatively highly prized fish, such as bluefish (*Pomatomus saltator*) and corvina (*Micropogonias furnieri*), which were sold during the same period and at the same location for R$10.00 per kg. However, restaurants did not show high demands for groupers according to our results (Additional file 1: Table S3), which could be due to the high prices observed in the market. Most commonly, these fishes are bought directly by the consumer at the fishery association of Copacabana of ‘Posto 6’. Market prices tend to follow demands (and the contrary is expected, i.e., a high demand increases the price, SUPP. MAT); however, high prices associated with low demand seem to be typical for declining species. This result could be an indirect form of evaluating the ‘fishing down the food web’, as suggested by Pauly et al. [101]. Moreover, the capture of small-sized individuals may not always reflect a declining population because, in the case of groupers, juveniles tend to be found in shallow waters where artisanal fisheries often obtain their catches, as shown here in Copacabana, Santos and São Sebastião (Fig. 5).

The distribution of *E. marginatus* is another important concern: did the species move southwards in Brazil? The distribution of dusky grouper was shown from the south of Brazil to southern Bahia by Froese and Pauly [22] and by Lopes et al. [15]; Condini et al. [21] also observed one individual in this area to the south of Bahia. Data from the IUCN (Fig. 2) and Craig et al. ([23], p., 187) show dusky grouper from southern to northeastern Brazil. Small-scale fishers from Bahia recognized the pictures (Fig. 7) but commented that the species was either rare or had not been observed for a long time. Therefore, some questions deserve more investigation, such as the following. a) Have dusky groupers disappeared from Bahia because of a decline in the population? b) Was the species always uncommon in Northeast Brazil? c) Did changes in water temperature maintain *E. morio* in Northeast Brazil but prompt dusky grouper to move southward? For *E. marginatus,* temperatures higher than 22–23° cause anomalous eggs (Dinis et al. [75], consulted October 14, 2018, at http://www.portaldoconhecimento.gov.cv/bitstream/10961/1531/1/Dinis’s%20paper.pdf). A recent study showed the importance of temperature in predicting the distribution of dusky grouper in Brazil, in addition to showing the reliability of using information from fishers to predict species distributions [15].

These are all important questions that we do not have enough data to answer or are only beginning to grasp their relevance through new statistical tools, which can be used to include ethnobiological data in more quantitative ways [15].

### Data on poor fisheries, key cultural species and local ecological knowledge

Despite the previous studies on the dusky grouper in Brazil (Tables 1 and 2), we observed that the data for this area are scattered and concentrated in a few regions. Condini et al. [21] showed that biological data were available from several dusky grouper fisheries, especially from Mediterranean countries such as Spain and Italy. For South America, these authors mentioned data from Santa Catarina (1988–2012).

Dusky grouper is a preferred food for small-scale fishers [69] and often seen as a delicacy. Moreover, the fish is recommended for consumption by ill persons along the Atlantic Forest coast [102]. Furthermore, the dusky grouper is a ‘noble fish’, i.e., small-scale fishers give it special regard as a target because it has a high value in the market. For 2016–2017, our data showed an average price of R$35.00 per kg for consumers who purchased the fish directly from the fishers at the Copacabana fishery.

In addition to cultural keystone species, the dusky grouper is also an ecological keystone species [21] that is currently classified as endangered by the IUCN Red List (https://newredlist.iucnredlist.org).

Our information on LEK is synthesized in Table 2 and shows early studies [2, 38, 103] that indicated that fish, crabs and mollusks (cephalopods) are important in the diet of the dusky grouper. Our results from interviews along the coast of Brazil (this study) also showed that crabs and mollusks were part of the dusky grouper diet according to the fishers. The same similarities (research results and fisher information) were found regarding the information on the habitat of the dusky grouper.

In Brazil, the dusky grouper is mainly caught using hook and line gear and spearfishing. Small-scale fishers fish relatively close to the shore (Fig. 5 and Begossi et al., [67]). This finding explains why size likely corresponds to small immature females or to a few mature females ([2, 8, 38]; this study) because juvenile fish often stay in shallow areas close to the shore [21]. The deep ranges are usually reached by small-scale fishers of Bahia state because the continental shelf is narrow in this part of Brazil (see [2, 38] for details.

Fishers also contributed some information on reproduction, which was said to occur during the spring and summer (autumn was also mentioned in the south of Brazil in this study), which was confirmed by other biological studies [21].

Groupers aquaculture is especially well developed in Asia: three countries account for approximately 92% of the global grouper production: China, Taiwan, and Indonesia [104]. Grouper aquaculture comprises approximately 47 grouper species and 15 grouper hybrids. Even though there are individual initiatives in Brazil, such as the Redemar Alevinos, visited in this study, we do not believe it will be possible to develop an ‘aquaculture of groupers in Brazil’. There are substantial technological gaps in Brazil compared to in Asia or the Mediterranean and enormous bureaucracies against research and innovation.

### Distribution and conservation

The distribution of *E. marginatus* between Rio de Janeiro (SE Brazil) and Bahia (NE Brazil) remains unknown. The State of Espírito Santo is located between Rio de Janeiro and Bahia, but it was not included in this study; additionally, we found no data published on landings from this area. In Bahia state (Figs. 2 and 10), fishers mentioned that this fish was rare and had ‘disappeared’. Condini et al. [21] registered an observation of this species in Bahia. We do not know whether *E. marginatus* was previously more abundant in Bahia (because fishers mentioned it) and then its population decreased or if the species moved southward due to environmental changes. It will be particularly important to investigate the water temperatures because the other species, *E. morio*, is more adapted to the warmer waters of Northeast Brazil; moreover, warm waters (i.e., above 22°) affect the egg development of *E. marginatus.*

Fishers did not know about the larvae of the dusky grouper and could not identify it in our samplings in Rio de Janeiro. Likewise, there is no information on dusky grouper larvae for the coast of Brazil [21]. However, during a study on the genetics of the dusky grouper along the southeastern coast of Brazil, Priolli et al. [35] concluded that a possible explanation for the genetic link of the populations of  Paraty and Rio de Janeiro could be the dusky grouper floating larvae, i.e., larval movements could be responsible for the genetic flow among the different islands of Paraty, reaching the coast of Rio de Janeiro (Copacabana). Schunter et al. [105] and Andrello et al. [48] emphasized the importance of understanding the population connectivity of the dusky grouper (such as by its larvae)  to protect the species. To answer how larvae flow and connect the populations in Brazil, systematic studies would need to be conducted to identify the presence of larvae with follow-up year-round monitoring at different sites.

### Local knowledge

In Brazil, many studies have reported on the local knowledge of groupers (Table 2), which is a type of data that could support conservation efforts. Lima et al. [106] found evidence of temporal changes in the Southeast Atlantic because fishers noticed that large-sized predators became scarce. A review of the literature focusing on conservation and management indicated that 16 studies explicitly provided data on *E. marginatus* that could aid conservation (Additional file 1: Table S4). When both these studies and the findings presented here are taken into account, the importance of suggesting very specific management measures is clear. For example, samplings from landings have shown that small-scale fisheries in Southeast Brazil have been catching groupers in the size range of 45–65 cm, which is above the minimum legal size (47 cm) [2, 38]. Small-scale fisheries have fishing spots for groupers around islands and reefs [35, 67]. Di Franco et al. [4] stressed the important role of coastal communities in the success of MPAs. Andrello et al. [48] identified the importance of understanding the behavior of dusky grouper larvae to analyze the connectivity among MPAs in the Mediterranean and showed that connectivity is low in the area but is key to sustaining recruitment within MPAs. Silvano et al. [73] showed that past fishing pressure might have pushed grouper fishing to more distant sites.

In Southeast Brazil, some studies have focused on small-scale fisheries, and others have specifically focused on the artisanal dusky grouper fishing [2, 8, 19, 38]. Priolli et al. [35] published one of the first studies on the population genetics of this species using samples collected from fisher landings from several sites around Paraty, Rio de Janeiro state, Southeast Brazil. The study concluded that only one population occurred in this area, which probably originated through genetic flow from larvae movement. In the Mediterranean, the genetics of dusky grouper species have been studied for many years [40, 44, 45]. Larval connectivity, thus, seems to be a key point linking populations in Brazil [35]; thus, connectivity is fundamental for conservation.

Despite our attempts, we did not obtain substantial information on larvae from small-scale fisheries along the coast of Brazil, and the scientific literature in this area did not provide information about dusky grouper larvae in this region [21]. However, small-scale fisheries have been useful in providing information on diet and habitat [8, 38, 57], migration [107] and reproduction (this study and Begossi et al. [2, 19]).

The MPAs in Brazil and Mediterranean areas are completely different. First, as mentioned above, there are data available on the dusky grouper from the Mediterranean, while the same is not true for Brazil. Second, MPAs appear to be better structured in the Mediterranean than in Brazil because they include zoning and enforcement of rules [47]. In Brazil, top-down processes are the rule, with scarce or no collaboration between researchers and fishers, and conflicts between local populations (and fishers) are common [9, 11–14]. Thus, we considered that a study based on the knowledge and experience from other areas around the world could provide insights into the conservation of this species in data-scarce fisheries, such as those in Brazil. Experiences from Latin American countries, where local ecological knowledge and/or citizen science were integrated into management programs (e.g., *E. morio* in Mexico and other *Epinephelus* species in Colombia and Panama) could work as examples to be followed in Brazil. Similar to these countries, Brazil also struggles with data scarcity, in addition to having a tradition of implementing top-down management. Thus, inspirational Latin American examples could be an opportunity for more participatory Brazilian MPAs. Our literature review indicated that the conservation of the dusky grouper could be improved by MPAs and by considering certain characteristics of this fish and its fisheries. For example, adults are sedentary and do not move long distances, and they are usually found in discrete spatial units with well-defined boundaries (i.e., islands or reefs), which are usually exploited and could be managed by local fisheries (Table 1). Furthermore, in some regions of the southeastern Brazilian coast, genetic analyses indicate the occurrence of a single large dusky grouper population [35], which may enhance the potential spillover and larval subsidy effects of well-chosen protected sites. However, there are two major constraints to the effectiveness of MPAs in protecting the groupers along the Brazilian coast. First, we lack detailed ecological information about the dusky grouper (e.g., habitat use, reproduction, population structure), which would be needed to select suitable areas to protect this fish. In the absence of these data, the choice of protected sites to be included in MPAs usually follow estimates or guesses by biologists, protection of other components of aquatic biodiversity (e.g., endemic plants, marine mammals, marine birds) or political considerations (e.g., enforcement and tourism). Second, because the dusky grouper is a commercial fish that usually occurs in populated regions of the coast, top-down government efforts to impose MPAs usually lead to severe socioecological conflicts with local fishers [11, 14].

### Marine protected areas

Although there is evidence of increased numbers of dusky grouper and other reef fish inside Brazilian MPAs [108–110], a remarkable case of conflicting and problematic MPAs involve the MPA of the Ecological Station of Tamoios in Paraty Bay on the southeastern Atlantic Forest coast. This MPA was arbitrarily established in a top-down approach without the consultation or consideration of local fishing communities. This MPA included some strictly protected islands, where fishing and even anchoring were banned close to and in the preferred fishing grounds of one fishing community. Thus, the MPA has not increased the fishing yields in the affected community close to its boundary, the densities of reef fish (including the dusky grouper) are not higher inside the MPA, and some islands located in the MPA are often exploited by fishers [11–14, 73]. Conversely, in the tropical Pacific, MPAs embedded in co-management systems that include local communities as partners have been effective in maintaining and increasing the abundance of exploited reef fish [111–113]. Therefore, we propose that efforts should be made to increase the cooperation among managers, researchers and local fishers to reach the ultimate goal of protecting the dusky grouper through the establishment of more effective and less conflicting MPAs on the Brazilian coast. This cooperation may greatly benefit from the detailed LEK that Brazilian coastal fishers have about the dusky grouper and other similar reef fish, which includes aspects of their feeding habits, trophic level (and contamination potential), habitat use and reproduction [8, 57, 114, 115]. For example, fishers have mentioned submerged rocky outcrops (locally called ‘lajes’ or ‘parceis’) as important habitats for the dusky grouper and other commercially important reef fish along the southeastern coast of Brazil [114]. These submerged habitats often include fishing grounds that are regularly used by these fishers [67], and at least some of these habitats could be included in the zoning and MPA systems [73].

Connectivity is also important. Studies on the genetics of *E. marginatus* from samples from Rio de Janeiro state concluded that one population occurred between the cities of Paraty and Rio de Janeiro (240 km distance). Larvae dispersal could be responsible for the gene flow between these areas [35]. In marine systems, pelagic larvae are especially important to this exchange [48, 105, 116].

Small-scale fisheries in Brazil target high-priced groupers by fishing with hooks and lines and spearfishing. Groupers are important fish to conserve as they are keystone species [21] and key ecological species; thus, special precautions need to be taken to accomplish conservation.

There are, shortcomings in the Brazilian environmental management system. These have included top-down processes when implementing MPAs, which have contributed to increase the suspicious of fishers about the impacts of MPAs upon their own communities.

In small-scale fisheries in Brazil, categories of conflicts include top-down processes in the implementation of MPAs [117], restriction on the uses of marine areas, with no consultation or participation of fishers (such as in the islands of Paraty bay, Rio de Janeiro [118]), and rejections by fishers of attempts in implementing extractive reserves by the governement, such as at Itaipu, Niteroi [119]. Recently, governmental agents took down the houses of local indigenous inhabitants, called *Caiçaras*, at the Ecological Station of Juréia-Itatins, at São Paulo (newspaper *Folha de São Paulo*, July 7, 2019).

Concerning the fishing of *E. marginatus,* legislation (*Portarias* 217, 445, 2018) has represented a problem to small-scale fishers, since they were forbidden to catch a very important species, a noble species, affecting their earnings.

There are, however, other initiatives in Latin America in which local ecological knowledge is more integrated to ecosystem-based management, and to MPAs: several examples are found in Orensanz et al. [120] and Baigun [121].

Actually, one of the fundamental steps in building up a legitimate process of fishery management should include the called “step zero” [122]: this should be the initial stage or process where ideas are communicated to stakeholders and also stakeholders are defined. The step zero is the stage where legitimacy could be built, avoiding top-down processes in the creation of a MPAs. The lack of success of many MPAs can be considered to be not due to lack of enforcement or monitoring, but due to lack of legitimacy and absence of “step zero”, provoking stakeholders to disbelief MPAs [122]. Fishers and other stakeholders often feel threatened by the establishment of MPAs and such reactions are often due to gaps in the implementation process; MPAs can be biologically successful, but can represent social failures [123]. Thus, establishing MPAs is more than a biological process, it embodies a political process: this can be at ends of a gradient, from government power to fishermen power. Government power represents most MPAs in Brazil, also because fishers in Brazil are poor, mostly illiterate or with low literacy, resulting in a state of disempowerment. Economic incentives could gain support of stakeholders and fishers in the establishment of MPAs [124]; suggestions for these incentives in Brazil were published [118]. Important to observe that grassroots movements, such as fishing agreements in the Amazon, have been more successful rather than imposed government reserves [117]. Finally, the lack of a representative process in Brazil regarding managing small-scale fisheries, associated with the lack of incentives (for example, economic incentives) and with the imposition of fishing restrictions (or of MPAs) through decrees, has helped to difficult the management of a vulnerable species, such as *Epinephelus marginatus.*

## Conclusions

The realities of Brazil and countries of the Mediterranean area are strikingly different. For example, in the Mediterranean, there is biological and ecological knowledge on groupers, and MPAs are constructed based on an array of studied factors; in contrast, in Brazil, the information is scarce, governmental authorities ignore the science [125], and protected areas are rarely constructed based on studies. In addition to these features, the protected areas in Brazil are established in a top-down manner and involve many conflicts with small-scale fisheries [9, 11, 14].

We should focus more directly on specific points to subsidize dusky grouper management:

1. Zoning: zoned regions should include areas where fishing is allowed for commerce, areas exclusively used for subsistence and no-take areas. Such zoning could be based on existing maps and helpful information from fishers. There are sound ecological examples to be followed in Brazil, such as the Sustainable Development Reserves (www.mamiraua.org; Castello [126]). Even fish such as *Arapaima gigas* (pirarucu) from the Mamiraruá reserve are certified, which is a rare case in Brazil.
2. Islands: because many grouper fishing spots are reefs, some or many of these locations are located on islands. Efforts to elaborate zoning to include reefs and islands could be undertaken. Fishers could help in the surveillance of these islands [118]. (Studies, such as that by Silvano et al. [73], should be enforced in this aspect).
3. Period of reproduction: the periods of grouper reproduction should be considered. Spring and summer are the reproduction periods that occur in Brazil. This study shows the reproduction periods in the different areas of Brazil, indicating that specific periods of suspended dusky grouper fishing could be established with the collaboration of fishers through the use of LEK. Naturally, we expect a collaborative system and not top-down approaches that have already been shown in other studies [69].
4. Larvae: the importance of larvae for gene flow among grouper populations is very relevant. Therefore, choices must be made regarding protected areas that are connected in some way, permitting gene flow.
5. Mapping: maps of habitats and maps for zoning within MPAs are both important. Groupers are sedentary reef fishes, which is a characteristic that facilitates the mapping of fishing spots used by fishers and zoning processes.
6. Finally, it is important to consider the local knowledge of small-scale fishers for data-poor fisheries. We illustrate and reinforce our conclusions by citing the review of Silvano and Valbo-Jørgensen ([127], p., 670), that indicate the detailed knowledge of fishers, that when recorded systematically can even help formulating new hypothesis that are tested using conventional research methods.

### An odd future: research difficulties in Brazil

Items 1 to 6 are of high importance in a country such as Brazil. However, our pessimistic scenario is that we cannot continue with research due to heavy penalties if the research protocol does not follow strict government orders. In addition to the lack of data on the dusky grouper and other important species, funding cuts and legislation in Brazil has made research very challenging. For example, to conduct studies on the dusky grouper, several authorizations must be obtained from governmental agencies (SISGEN, SISBIO – Decree 8722 from May 11, 2016, among others), as well as from a university ethics committee, making it sometimes impossible due to the need to wait several months, or more than a year, to conduct research. Additionally, researchers can now incur high monetary penalties if any requested information is not reported to governmental authorities (including any knowledge concerning a living creature). Such policies have driven researchers to restrict their own research agenda to avoid any issues. The data shown here utilized the SISBIO and SISGEN protocols under the numbers 53,824 and AB53669, respectively. Unfortunately, we do not intend to continue studies because the bureaucracy is extensive and the penalties are high. Azevedo-Santos et al. [125] commented on the lack of communication between scientists and policymakers that was historically considered deficient. Scientists are rarely consulted or heard, and they are currently under scrutiny by the federal government. Unfortunately, this decree could force researchers to continue studies on nonnative species or in other countries.

### Supplementary information

**Additional file 1: Table S1.** Local Ecological Knowledge with total (number) of interviewees. **Table S2.** Features of small-scale fishers interviewed (age, time fishing and time of residence in the place). **Table S3.** Restaurants visited in the places studied. **Table S4.** Conservation efforts: selected literature on the dusky grouper, *Epinephelus marginatus*, 2010_2017 (alphabetic and year order). **Table S5.** Selected earlier studies and observations on Brazil on dusky grouper (*Epinephelus marginatus*) and red grouper (*E. morio*) of small-scale fisheries (Begossi and Figueiredo, 1995 [128]; Begossi et al., 2010: 70–72; 86 [129]; Begossi et al., 2013:137 [67]; Camargo and Begossi, 2013:122–127 [130]), Lopes et al. (2010, 253) [131]; Ramires et al., 2015 [102]; Begossi et al., 2016 [2]).

## Figures and Tables

**Fig. 11:**
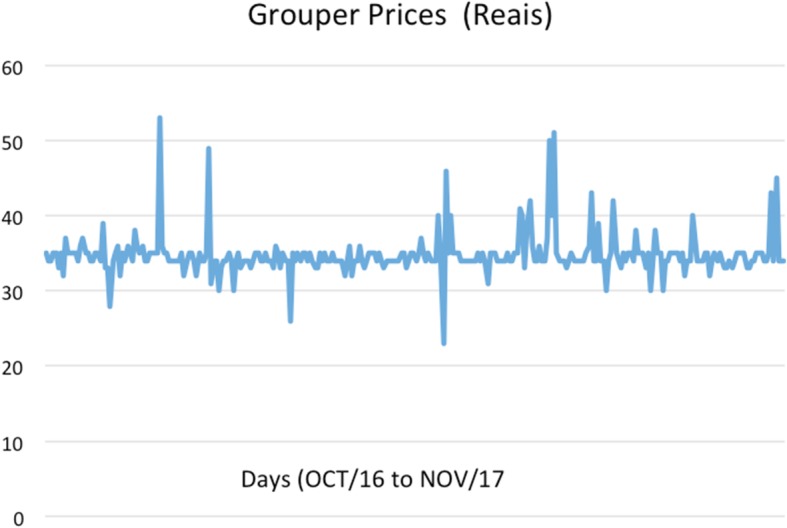
Prices per kg of dusky grouper at the fishery of Copacabana, Rio de Janeiro, from October 12, 2016 to November 7, 2017 (300 days, samples per month). Exchange rates of dolar: October 12, 2016=3,20 reais; November 7, 2017=3,27 reais)

**Fig. 12:**
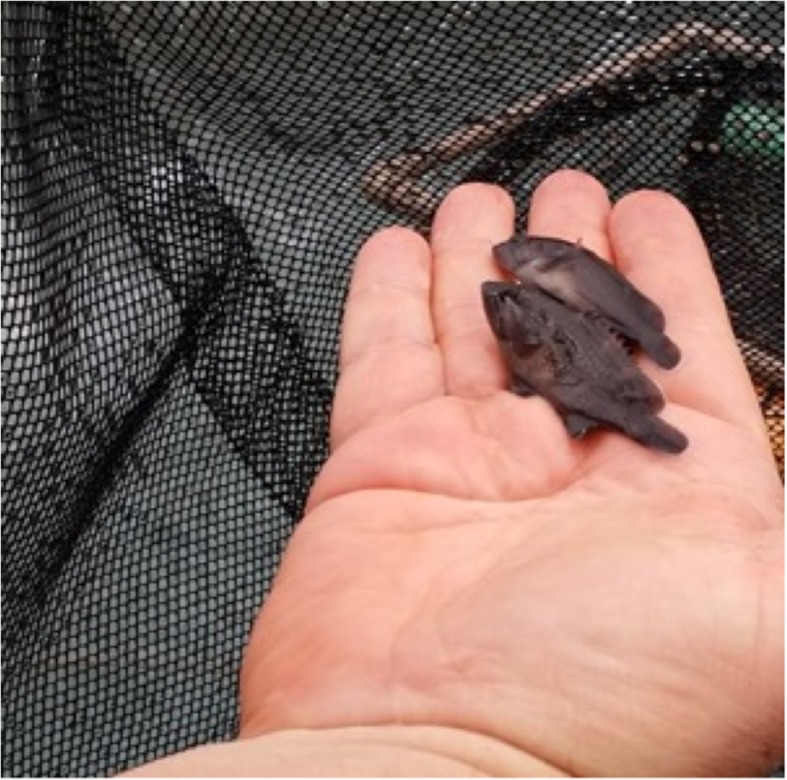
Juvenile *E. marginatus* from aquaculture (Redemar Alevinos), Ilhabela (February 2017)

## Data Availability

Data are available at the Fisheries and Food Institute Arquives.
