# Supplementary material for: Fishers and groupers (Epinephelus marginatus and E. morio) in the coast of Brazil: integrating information for conservation
Source: J Ethnobiol Ethnomed. 2019 Nov 6;15:53. doi: 10.1186/s13002-019-0331-2 (PMC6836445; doi:10.1186/s13002-019-0331-2)
Supplement: Supplementary file 1 — Additional file 1: Table S1. Local Ecological Knowledge with total (number) of interviewees. Table S2. Features of small-scale fishers interviewed (age, time fishing and time of residence in the place). Table S3. Restaurants visited in the places studied. Table S4. Conservation efforts: selected literature on the dusky grouper, Epinephelus marginatus, 2010_2017 (alphabetic and year order). Table S5. Selected earlier studies and observations on Brazil on dusky grouper (Epinephelus marginatus) and red grouper (E. morio) of small-scale fisheries (Begossi and Figueiredo, 1995 [128]; Begossi et al., 2010: 70–72; 86 [129]; Begossi et al., 2013:137 [67]; Camargo and Begossi, 2013:122–127 [130]), Lopes et al. (2010, 253) [131]; Ramires et al., 2015 [102]; Begossi et al., 2016 [2]). [file 13002_2019_331_MOESM1_ESM.doc]

**SUPPLEM. MATERIAL**

**FISHERS AND GROUPERS**

**Table 6**. Local Ecological Knowledge with total (number) of

interviewees

| **State** | **Place** | **Local** | **Number of interviewees** | |
| --- | --- | --- | --- | --- |
| BAHIA (BA) | NE Bahia | Arembepe | 3 | 10 |
|  |  | Porto Sauipe | 5 |  |
|  |  | Praia Forte | 2 |  |
|  | SE Bahia | Acuípe | 2 | 15 |
|  |  | Ilheus Pontal | 5 |  |
|  |  | Itacare z-18 | 5 |  |
|  |  | Pedras de Una | 3 |  |
| RIO DE JANEIRO (RJ) | Copacabana | Posto 6 | 11 | 11 |
| SÃO PAULO (SP) | Bertioga |  | 3 | 3 |
|  | Guaruja | Praia do Goes | 2 | 12 |
|  |  | Praia do Guaiúba | 1 |  |
|  |  | Santa Cruz dos Navegantes | 9 |  |
|  | Santos |  | 2 | 2 |
|  | São Sebastião | Baía do Araçá | 2 | 8 |
|  |  | Porto Grande | 3 |  |
|  |  | Santiago | 1 |  |
|  |  | São Francisco | 1 |  |
|  |  | Toque-Toque Pequeno | 1 |  |
| SANTA CATARINA (SC) | Florianópolis | Pantano do Sul | 10 | 10 |
| **Total** |  |  |  | **71** |

**Table 7**. Features of small-scale fishers interviewed (age, time fishing and time of residence in the place)

| **Local** | **Age** | | **Years Fishing** | | **Years of Residence** | |
| --- | --- | --- | --- | --- | --- | --- |
|  | **Mean** | **StDev** | **Mean** | **StDev** | **Mean** | **StDev** |
| NE BAHIA (BA) | 58.1 | 7.56 | 35.9 | 13.03 | 42.9 | 18.79 |
| RIO DE JANEIRO (RJ) | 49.4 | 14.01 | 31.4 | 15.44 | 41.3 | 18.12 |
| SANTA CATARINA (SC) | 57.3 | 15.15 | 31.4 | 11.96 | 42.4 | 16.18 |
| SÃO PAULO (SP) BERTIOGA, SANTOS, GUARUJÄ | 54.7 | 13.53 | 28.6 | 13.12 | 39.6 | 21.65 |
| SÃO PAULO (SP): S. SEBASTIÃO | 55.3 | 16.78 | 39.1 | 16.44 | 39.5 | 19.02 |
| SE BAHIA (BA) | 50.7 | 13.55 | 31.2 | 10.53 | 37.6 | 13.56 |
| **TOTAL** | **53.9** | **13.48** | **32.2** | **13.18** | **40.3** | **17.59** |

Table 8. Restaurants visited in the places studied.

| **Site/date**  **(municipality)** | **Name / no. restaurants** | **Fish/kg/month (average)**  ***Buyer** |  | **Fish on menu** |
| --- | --- | --- | --- | --- |
| **State: Bahia** |  |  |  |  |
| PORTO DO SAUÍPE (Entre Rios) | Dois Irmãos  Peixe na Brasa  2 | 11  *Fishery | 3  No | Agulhinha, corvina (sand drum), enchova (bluefish), pescada (weakfish), pititinga, sardinha (sardine), vermelho (snapper) |
| PRAIA DO FORTE  (Mata S. João)  (11/10/16) | Recanto  Barra Sapiranga  2 | 315  *Fishery | 18  No | Atum (tuna), dourado, guaricema, olho de boi, vermelho (snapper) |
| AREMBEPE  (Camaçari)  11/11/16 | Neusa  Coló  2 | 40  *Fishers | No  No | Arraia (ray), badejo (*Mycteroperca/*grouper), cavala, dourado, vermelho (snapper) |
| ILHÉUS, Pontal  (Ilhéus)  11/12/16 | Bar do Mico  Enseadas  2 | 105  *Fishers (Z-19) | No  No | Dourado, guaíuba, olho de boi, pescada (weakfish), vermelho (snapper) |
| ACUÍPE  (Ilhéus)  11/13/16 | Bar do Coringa  1 | 197  *Fishers | 24 | Arraia (ray), badejo *(Mycteroperca*/grouper), cação (shark), dourado, guaiúba, pescada (weakfish), robalo (snook), tainha (mullet), vermelho (snapper) |
| PEDRAS DO UNA  (Una)  11/13/16 | Bar da Bete  1 | 32 | No | Arraia (ray), bagre (catfish), garapeba, robalo (snook), tainha (mullet). |
| ITACARÉ  (Itacaré) | No Boteco  Mediterrâneo  2 | 340  *Fishers | No | Badejo *(Mycteroperca*/grouper), cavala (Scombridae), dourado, guaiúba, olho de boi, vermelho (snapper). |
| **State: Rio de Janeiro** |  |  |  |  |
| COPACABANA  (Rio de Janeiro)  11/29/16  1/2/17 | Venga  Pigale  Umesusai  No mangue  4 | 650  *Fishers (Venga) | No  No  No  Few | Olho de cão, pargo, pescada (weakfish), robalo (snook) |
| **State: São Paulo** |  |  |  |  |
| S. SEBASTIÃO  10/21/16  11/28/16 | Sem compromisso  Família  2 | 300  *Fishers | 55 | --- |
| CARAGUATATUBA  12/2/16 | Toca do robalo  1 | 600  *Fishers | 300 | -- |
| SANTOS  (Ponta da Praia)  1/12/17 | Mar del Plata  Ki delícia  2 | 275  Several (Mar del Plata) | 200  No | Badejo *(Mycteroperca*/grouper), garoupa (dusky grouper), cação (shark), linguado (flounder), merluza, pescada (weakfish), salmão, tainha (mullet) |
| GUARUJÁ  (Center and Perequê)  1/12-13/17 | Bambuzal  Céu Azul  O Pescador  Caiçara  4 | 632 | No  No  No  No | Badejo *(Mycteroperca*/grouper), cação (shark), corvina (sand drum), pangassium, pescada (weakfish), salmão, tainha (mullet) |
| PRAIA GRANDE  1/11/17 | Petisco da Guilhermina  Terraço Paris  2 | 106 | No  No | Badejo *(Mycteroperca*/grouper), linguado (flounder), merluza, pescada (weakfish), porquinho, salmão, tainha (mullet) |
| **State: Santa Catarina** |  |  |  |  |
| Pântano do Sul,  Florianópolis  3/8/17 | Canoa grande  Arante  2 | 1,250  Fishers | No  30 | Bacalhau (cod), enchova (bluefish), garoupa (dusky grouper), tainha (mullet) |
| **TOTAL** | 29 | Average = 347 kg/mes  Total = 4,853 kg |  |  |

Table 9. Conservation efforts: selected literature on the dusky grouper, *Epinephelus marginatus*, 2010_2017 (alphabetic and year order).

| **Location** | **Subject** | **Reference** |
| --- | --- | --- |
| BRAZIL (SE) | CONSERVATION/FISHERY. Fish consumption, prohibition to catch several species. Small-scale fisheries reaction, since many are species consumed, including dusky grouper. Conflicts with small-scale fishers and threatened to current jobs. The decree by the government bounces back and forth due to dubious legalities and lack of studies. | Begossi et al. (2017) |
| Worldwide | BIOLOGY. Review on the biology and on the literature of dusky grouper. Suggestions for management of the species, as follows: (1) a minimum size limit for capture (2) a limitation in the number of fishing vessels (3) a total ban (4) a system of catch quotas (5) a fishing ban during specific periods (spawning seasons and spawning aggregations) (6) the creation of marine protected areas (MPAs). | Condini *et al*. (2017) |
| BRAZIL (S) | BIOLOGY/ECOLOGY. Biological and ecological features (body size, age, diet and trophic position) to investigate mercury concentration in dusky groupers. Location: rocky bottoms along the Southern Brazilian coast. Number: 244 dusky groupers analyzed. Relatively high mercury concentrations; possible cause: proximity to pollution sources associated with human activities. Large individuals (650 mm and 8 years old) with mercury contamination levels potentially harmful for fish species and above threshold for human consumption. | Condini *et al*. (2016) |
| BRAZIL (S) | BIOLOGY. Age and growth of dusky grouper (analysis of otoliths). Location: Carpinteiro Bank (Southern Brazil, Atlantic). Number: 211 groupers. Ages 1-40 years old; most fish 2-8 years old. Von Bertalanffy growth parameters for pooled sexes were [L.sub.[infinity]]=900.9 mm, K=0.129 and [t.sub.0]=-1.45. Offshore fish generally older than inshore fish. Fishery management should ensure dusky groupers of Carpinteiro Bank. | Condini et al. (2014) |
| BRAZIL (SE) | FISHERY/ECOLOGY. Sampling on landings: 21 months, 800 individuals of dusky grouper examined. Location: Copacabana, Rio de Janeiro, Brazil. Mean length of dusky grouper caught by fishers (N = 796): 52 cm. Groupers ranged 45-65 cm, above the minimum allowed size in Brazil (47 cm) and above the size at first maturity (range of 35-60 cm for females). | Begossi et al. 2016 |
| BRAZIL (SE) | GENETICS/ECOLOGY. Genetic, microsatellite markers of *Epinephelus marginatus* populations collected from artisanal fisheries sites (fishing spots) in southeastern Brazil. Location: Paraty and Rio de Janeiro (Copacabana), Rio de Janeiro State, Brazil. Number: 122 muscle from dorsal fins (Paraty) and 30 from (Copacabana).The microsatellite loci showed no population subdivisions. All samples from different geographical sites shared the same genetic structure. The effective population size (Ne) resulted in 663 individuals between the Paraty (RJ) and Rio de Janeiro (RJ) populations. | Priolli *et al*. (2016) |
| COAST OF BRAZIL  Mucuripe, Fortaleza, Ceará (NE Brazil) to Pantâno do Sul, Florianópolis, Santa Catarina State.  Dusky grouper: coasts of São Paulo and Rio de Janeiro. | FISHERIES/ECOLOGY. Fishing spots for fishing dusky groupers were mapped based on information from artisanal fishers in the coasts of Rio de Janeiro and São Paulo. Management suggestion:  - coastal zones based upon informal fishing rights of local fishers (rights relative to informal ownership of fishing spots, per area or per community). | Begossi et al. (2013) |
| COAST OF BRAZIL Mucuripe, Fortaleza, Ceará to Pantâno do Sul, Florianópolis, Santa Catarina State. | Distribution of Serranidae (fish collected and observations), including dusky grouper. Number: 1,761 fish landings sampled; 1,453 fish collected from these landings; 585 fishers interviewed from 14 fishing communities on the northeastern, southeastern, and southern Brazilian coast. The number of grouper species collected, from different genera included 16 species. Management suggestion: with the support or collaboration of fishers, managing reef fish stocks could gain success. Fishers could monitor reef fishes located close to or in their fishing spots; economic incentives could help in the interaction fisher-management. | Begossi et. al. (2012) |
| PARATY, RJ, BRAZIL (SE) | FISHERIES/ECOLOGY. Sampling on landing points included 220 groupers caught by fishers. Results showed Spring (September-December) as the main fishing season of groupers. Most groupers shown a total length of less than 50 cm. Visible eggs were not observed. The volume of the gonads ranged from 0.5-2.5ml. One-half of the grouper stomachs analyzed in this study were empty. Crabs represented 35% of the contents of the non- empty stomachs, and 15% fish.  Management suggestions:  *a)* Due to the importance of dusky grouper in the commerce and consumption of small- scale fishers, encouragement mechanisms are needed to have fishers participating in the protection of this species;  b) Mechanisms that already exist in the Brazilian legislation (*defeso*) along with PES (payments for environmental services) are suggested (see specific publication in this aspect, Begossi et al., 2011).  c) Improving existing MPA design is important, such as the focus in the protection of target reef fish. Consultation with local fishers, can integrate management schemes as done for the Amazon small-scale fisheries (see Sustainable Development Reserves in Brazil, such as Mamirauá, Amazonas State,  <https://www.mamiraua.org.br/> | Begossi et al. (2012) |
| SE BRAZIL | FISHERIES/ECOLOGY. Effects of fishing pressure on the density of three reef species, including dusky grouper. Investigations covered 21 islands in the coast of Paraty, Rio de Janeiro State. Among these, 15 outside the MPA and 6 inside the MPA.  Fish densities were recorded in number and biomass. The density of dusky grouper increased with the island distance from one of the fishing villages. Past fishing could have decreased the abundance of this species.  Management suggestion: a redefinition of MPA boundaries to reconcile fish conservation, fishing activities and the food security of fishers. | Silvano et al. (2017) |
| SE BRAZIL | BIOLOGY. The scales of 135 dusky groupers were examined.  The coefficient of natural mortality estimated is M=0,204; the low growth coefficient is K=0,075; high life expectancy observed (T_max_= 37,7 years); carnivore species, 5^th^ trophic level. | Ximenes-Carvalho et al. (2012) |
| BRAZIL (SE) | CONSERVATION: Paraty bay; conflicts over closure of islands for small-scale fisheries (residents) at Paraty. Suggestion of PES (payment for environmental services). | Begossi et al. (2011) |
| South of BRAZIL Rio Grande, Rio Grande do Sul State. | BIOLOGY. Age structure, growth and reproduction of individuals: 130 individuals observed, length ranging from 260 -800 mm. The current L50 estimate of 451.3 mm indicates that most individuals captured in this area are immature. | Seyboth et al. (2011) |
| Laboratory | BIOLOGY. Experiments: during the spring, AI promoted sex change after 9 weeks and the sperm produced was able to fertilize grouper oocytes. During the summer, the sex change was incomplete; | Garcia et al. (2013) |
| MEDITERRANEAN  two MPAs (one in the Balearic Islands and one in Sardinia) | ECOLOGY. Evaluation of connectivity between MPAs in the Mediterranean Sea. Approximately a hundred MPAs exist in that area.  Features: high heterogeneity of MPA distribution, low density in the South-Eastern Mediterranean, mean larvae dispersal distance of 120 km. This means 20% of the continental shelf without larval supply. Low connectivity.  Management suggestions: new challenges for a network of well-connected MPAs that could provide recruitment to the whole area. | Andrello et al. (2016) |
| MEDITERRANEAN | ECOLOGY. Location: Mediterranean Sea were MPAs are numerous (30). MPAs with good reinforcement triggers effective recoveries. Analysis on new MPAs that could be established in the Mediterranean Sea | Guidetti et al. 2014 |
| MEDITERRANEAN | FISHERIES/ECOLOGY. Ecological, social and economic attributes of small-scale fisheries from 25 Mediterranean MPAs. Results: fish stocks are healthier, fishermen incomes are higher and the social acceptance of management practices is better if fishermen engagement in MPA management occurs; key findings for the Mediterranean coastal communities and the sustainability of fisheries and of the resources. | Di Franco et al. (2017) |
| ITALY (Sardinia) | FISHERIES/ECOLOGY. Habitat mapping and its importance for management and conservation. Importance of maps for: habitats, development pressures, tourist facilities or protected areas. Application mapping methodology to the case study of the marine protected area ‘Tavolara Punta Coda Cavallo’, Sardinia. | Rovere *et al*. (2013) |

**Table 10.** Selected earlier studies and observations on Brazil on dusky grouper (*Epinephelus marginatus*) and red grouper (*E. morio*) from small-scale fisheries (Begossi and Figueiredo, 1995; Begossi et al., 2010: 70-72; 86; Begossi et al., 2013:137; Camargo and Begossi, 2006:122-127), Lopes et. al. (2010: 253); Ramires et al., 2015; Begossi et al., 2016)

| Locality | Year of study (Presence in landings, consumption, commerce, and cultural importance) | Species |
| --- | --- | --- |
| Buzios Island (São Paulo) | 1986-87: consumed and used in illness | *E. marginatus* and secondarily *E. morio* |
| Sepetiba bay (Rio de Janeiro) | 1990-91: consumed and sold | *E. marginatus* and secondarily *E. morio* |
| Grande Island and Paraty | 2010_highly cited in interviews as consumed and sold (413 fishers interviewed) | *E. marginatus* |
| Fishing spots from Bertioga São Paulo) throughout Paraty beach (Rio de Janeiro), along the coast | 1986-2003 | *E. marginatus* |
| Copacabana (Rio de Janeiro | 2013-2016 | *E. marginatus* |
| Ilhabela (São Paulo) | 2005-2007: Local knowledge | *E. marginatus* and *E. morio* |
| Coast of Brazil (Bahia, Rio de Janeiro, Santa Catarina) (92 interviews) | 2004-2005 | *E. marginatus* and *E. morio* |
